# Global burden of cardiovascular disease attributable to lead exposure: based on the global burden of disease study 2021

**DOI:** 10.3389/fpubh.2026.1690287

**Published:** 2026-02-27

**Authors:** Zhang Lin, Zhongwu Zhang, Qingxin Zeng, Song Luo, Jin Xu

**Affiliations:** 1 Department of Geriatrics, Fujian Provincial Geriatric Hospital, School of Clinical Medicine, Fujian Medical University, Fuzhou, China; 2 Department of Cardiology, Fujian Provincial Geriatric Hospital, School of Clinical Medicine, Fujian Medical University, Fuzhou, China; 3 Department of Gastroenterology, Fujian Provincial Geriatric Hospital, School of Clinical Medicine, Fujian Medical University, Fuzhou, China

**Keywords:** cardiovascular-disease, decomposition analysis, frontier analysis, global burden of disease, lead exposure, socioeconomic inequality

## Abstract

**Background:**

Lead exposure is an important but under-recognized environmental contributor to cardiovascular disease (CVD). Using GBD 2021, we quantified long-term trends, socioeconomic inequalities, key drivers, prevention potential, and future trajectories of lead-attributable CVD burden.

**Methods:**

We extracted GBD 2021 estimates (1990–2021) for deaths, DALYs, and age-standardized rates (ASMR/ASDR) by sex, SDI quintile, region, and country. Temporal trends were summarized using estimated annual percentage change. We applied Das Gupta–type decomposition (population growth, aging, epidemiologic change), assessed inequality using slope index of inequality and concentration index, evaluated efficiency gaps via SDI-based frontier (LOESS), and projected ASMR/ASDR to 2040 using ARIMA models.

**Results:**

Globally, ASMR and ASDR declined from 1990 to 2021 (EAPC −0.76%/year for ASMR; −1.09%/year for ASDR), yet the absolute burden remained high in 2021 (≈1.48 million deaths; ≈30.0 million DALYs), with higher counts in males. High-SDI settings achieved the fastest rate reductions, whereas low-SDI regions experienced increasing deaths/DALYs and slower declines. Decomposition showed population growth was the dominant driver of increasing deaths (96.07%) and DALYs (131.44%), partially offset by favorable epidemiologic change (−43.19% deaths; −78.83% DALYs). Inequality widened from 1990 to 2021 (ASMR SII − 2.62 to −7.15; ASDR SII − 70.24 to −144.88; concentration indices became more negative). Frontier analysis identified large efficiency gaps in many low- and middle-SDI countries. Projections suggest continued declines in age-standardized rates to 2040.

**Conclusion:**

Despite falling age-standardized rates, lead-attributable CVD burden remains substantial and increasingly concentrated in lower-SDI populations, driven mainly by population growth and aging. SDI-stratified policies combining lead source control with scalable CVD prevention are essential to reduce inequities and close efficiency gaps.

## 1 Introduction

Lead exposure is a well-recognized environmental risk factor for cardiovascular disease (CVD) (1). However, its contribution to the global cardiovascular disease burden remains insufficiently appreciated. Within the Global Burden of Disease (GBD) comparative risk assessment framework, cardiovascular diseases in this study primarily include ischemic heart disease (IHD) and stroke, which together account for the vast majority of global CVD-related deaths (2). The latest estimates from the GBD database indicate that deaths and disability-adjusted life years (DALYs) attributable to lead exposure are substantially higher than previously recognized, with the problem being particularly severe in low- and middle-income countries (LMICs) due to rapid industrialization and weak environmental regulations that have led to persistent and even worsening lead pollution (3, 4). Although population blood lead levels have markedly declined in high sociodemographic index (SDI) regions as a result of stringent environmental policies, the cardiovascular burden associated with lead exposure remains a major global public health concern.

An increasing body of experimental and epidemiological evidence suggests that lead exposure adversely affects cardiovascular health through multiple biological pathways, including oxidative stress, endothelial dysfunction, inflammatory responses, and epigenetic regulation (5, 6). Beyond overt cardiovascular events, recent population-based studies have demonstrated significant non-linear associations between blood lead levels and subclinical myocardial injury even among individuals without clinically diagnosed CVD, highlighting the latent cardiovascular toxicity of low-dose lead exposure (7, 8). These findings underscore the importance of assessing the long-term cardiovascular burden attributable to lead exposure across diverse populations.

With ongoing global population aging and epidemiological transition, the distribution patterns of lead exposure-attributable CVD have undergone marked changes. For example, ischemic heart disease has gradually replaced stroke as the leading cause of cardiovascular death attributable to lead exposure, accounting for more than 40% of the global CVD burden (9). However, this shift in disease composition varies substantially across SDI regions. High-SDI regions have achieved a rapid decline in age-standardized mortality rates (ASMR) through effective pollution control and cardiovascular prevention strategies, whereas low-SDI regions face multiple challenges arising from population growth, aging, and limited environmental governance capacity (10). Therefore, understanding how demographic changes, epidemiological transition, and socioeconomic development jointly influence the burden of lead exposure-attributable CVD is essential for developing targeted and equitable intervention strategies.

Previous research has shown a pronounced socioeconomic gradient in the burden of lead exposure-attributable CVD, with low-SDI populations bearing more than 60% of the global DALY losses (11). This health inequality reflects both the unequal distribution of environmental lead pollution and disparities in healthcare system performance and disease control capacity among regions. Although frontier analytical approaches have been increasingly employed to evaluate health system performance and disease prevention efficiency (12), their application in exploring the prevention potential of diseases associated with environmental risk factors remains limited. Moreover, while population growth and aging are widely recognized as major drivers of the global increase in cardiovascular burden (13), the relative contributions of these factors to lead exposure-attributable CVD have not yet been systematically quantified.

Most existing studies on lead exposure-attributable CVD have focused on burden estimation at specific time points, with limited attention to long-term trends, driving forces, socioeconomic inequalities, and future prevention potential. Using data from the GBD 2021 study, the present research integrates decomposition analysis, health inequality assessment, frontier analysis, and time-series forecasting to comprehensively evaluate the burden of CVD attributable to lead exposure. The specific objectives of this study are: to quantify the contributions of population growth, aging, and epidemiological changes to the global and regional burden of lead exposure-attributable CVD from 1990 to 2021; to assess socioeconomic inequalities and efficiency gaps in lead exposure-attributable CVD prevention across SDI regions; and to project the burden trends of lead exposure-attributable CVD from 2021 to 2040. By elucidating the complex interplay between lead exposure, cardiovascular disease, and the social determinants of health, this study aims to provide scientific evidence to support the development of equitable environmental governance policies and targeted cardiovascular disease prevention strategies at the global level.

## 2 Materials and methods

### 2.1 Data sources and study variables

We conducted a retrospective secondary analysis using estimates from the Global Burden of Disease (GBD) 2021 study, which provides internally consistent cause- and risk-specific burden estimates for 204 countries and territories across 21 GBD regions from 1990 onward (14). Data were extracted from the Global Health Data Exchange (GHDx) Results tool.^1^ For this study, the risk factor of interest was lead exposure and the outcomes were cardiovascular disease (CVD) burden attributable to lead exposure under the GBD comparative risk assessment framework (15). We extracted lead-attributable estimates by year (1990–2021), location (global, SDI quintiles, regions, and countries/territories), sex, and age group, including deaths, DALYs, and—where applicable—the DALY components YLLs and YLDs, as well as corresponding age-standardized rates (ASMRs and ASDRs) (14). Uncertainty was quantified using 1,000 posterior draws and summarized as 95% uncertainty intervals (UIs) (14).

### 2.2 Decomposition analysis

We performed demographic decomposition to quantify the relative contributions of three drivers to changes in lead exposure-attributable CVD burden between 1990 and 2021: (1) population growth, (2) population aging (changes in age structure), and (3) epidemiological change (changes in age-specific rates independent of demography) (16). The decomposition was implemented using a Das Gupta–type approach to partition temporal changes while holding other components constant. Uncertainty was propagated by repeating the decomposition across 1,000 GBD draws and summarizing 95% UIs from the empirical draw distribution (16).

### 2.3 Health inequality analysis

Socio-demographic Index (SDI) is a composite indicator of development status in the GBD framework, calculated as the geometric mean of lag-distributed income per capita, mean educational attainment (age ≥15 years), and total fertility rate under age 25, scaled from 0 to 1 (17). Countries and territories were categorized into five SDI quintiles (low, low-middle, middle, high-middle, and high). To assess absolute inequality, we modeled the association between SDI and lead exposure-attributable CVD burden using robust regression (RLM) at the country/territory level to reduce sensitivity to outliers and heteroskedasticity. Consistent with typical GBD inequality presentations, the dependent variables were the age-standardized rates of lead exposure-attributable CVD burden (ASMR and ASDR; and when applicable, age-standardized YLL and YLD rates), and the independent variable was SDI (18). The model was specified as: ASR = *α* + *β* × SDI + *ε*, with countries/territories weighted by population size. The fitted regression curves and slope coefficients (β) were used to quantify changes in absolute inequality over time (1990 vs. 2021). To assess relative inequality, we constructed Lorenz curves and calculated concentration indices by ranking countries/territories by SDI and integrating the area between the Lorenz curve and the line of equality, following standard concentration index methodology (18).

### 2.4 Frontier analysis

To evaluate prevention potential and efficiency gaps across development levels, we constructed an SDI-based frontier model in which SDI was the predictor and lead exposure-attributable CVD age-standardized burden rates (ASMR and ASDR) were outcomes. Frontier analysis identifies the theoretical minimum burden achievable at a given SDI level and uses this benchmark to quantify an efficiency gap (i.e., the distance between observed burden and the frontier). The optimal performance boundary was fitted using locally weighted regression (LOESS) (19). We conducted sensitivity checks across smoothing spans (0.3–0.5) and generated 95% UIs using 1,000 bootstrap iterations (19). The efficiency gap was defined as the absolute difference between each country/territory’s observed ASR and the SDI-specific frontier estimate, with larger gaps indicating greater potential for burden reduction under current development conditions. This structure follows the standard frontier presentation commonly used in Frontiers-style GBD studies.

### 2.5 Time-series projections

We used autoregressive integrated moving average (ARIMA) models to project future trends in lead exposure-attributable CVD burden from 2021 to 2040 for key age-standardized rates (ASMR and ASDR; and where applicable, age-standardized YLL and YLD rates). Stationarity was evaluated using the Augmented Dickey–Fuller test, and ARIMA parameters (p, d, q) were selected based on autocorrelation/partial autocorrelation patterns and information criteria (AIC/BIC) (20). Residual autocorrelation was assessed using the Ljung–Box test. Model projections were generated for each outcome time series, with uncertainty characterized by model-based prediction intervals.

### 2.6 Statistical analysis and data visualization

We summarized lead exposure-attributable CVD burden by year, location, SDI quintile, sex, and age group using counts (deaths, DALYs, YLLs, YLDs) and age-standardized rates per 100,000 population. Temporal trends in age-standardized rates were quantified using estimated annual percentage change (EAPC) from log-linear regression: ln(ASR) = *α* + *β* × year + *ε*, with EAPC = 100 × (exp(β) − 1) (21). Trend direction was classified as increasing (EAPC and 95%CI lower bound > 0), decreasing (EAPC and 95%CI upper bound < 0), or stable (95%CI spanning 0), consistent with common GBD reporting practice (14, 22, 23). All analyses were conducted in R (version 4.4.2). Data visualization was implemented using ggplot2 and the JD_GBDR (v2.5.4) (24).

## 3 Results

### 3.1 Global burden

**Figure 1:**
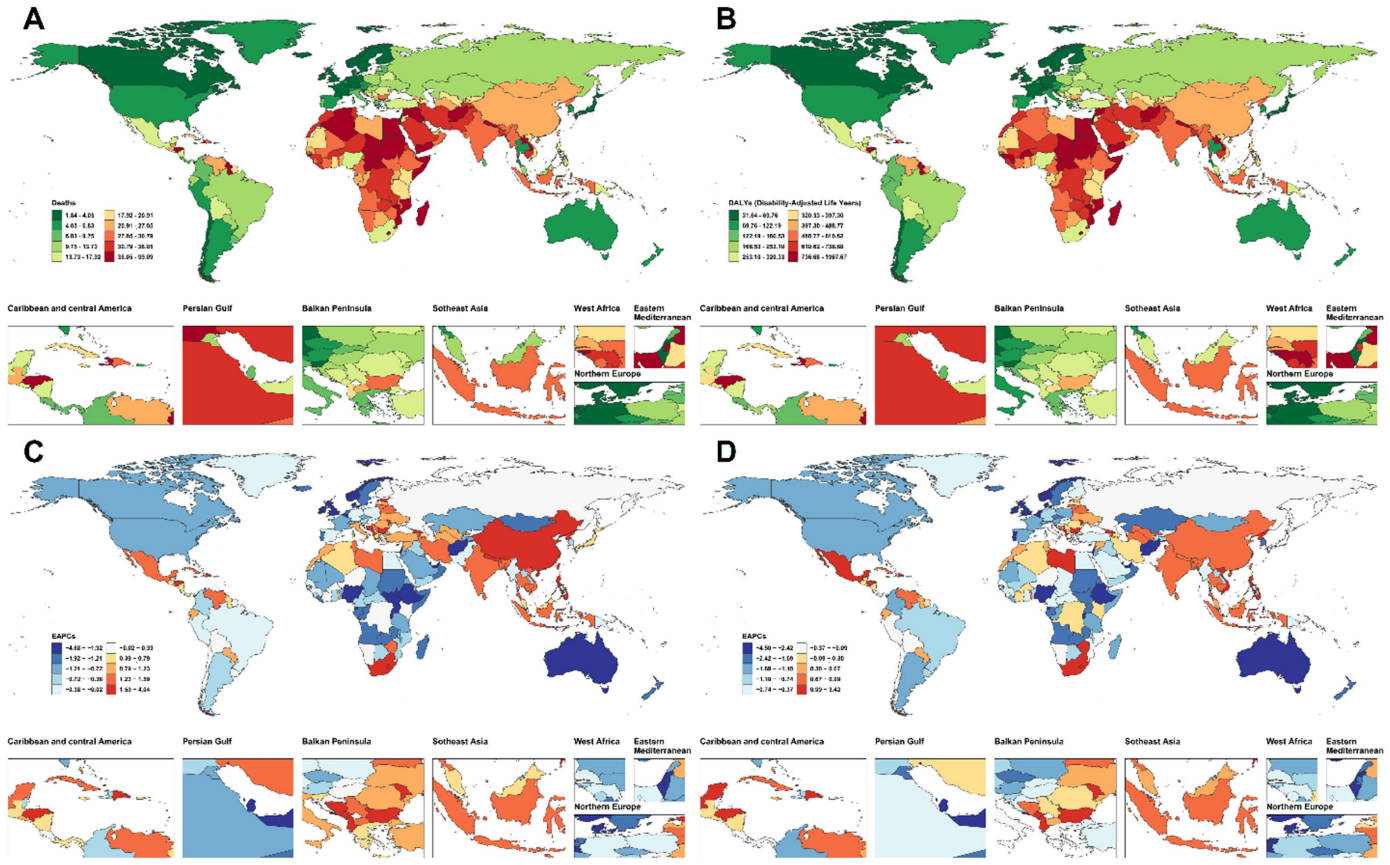
Age-standardized mortality rate (ASMR) and age-standardized death risk (ASDR) of CVD attributable to lead exposure across global regions at different time points, with corresponding estimated annual percentage changes (EAPC). **(A,B)** Geographic distribution of ASMR and ASDR; **(C,D)** EAPC trends for ASMR and ASDR.

From 1990 to 2021, the global ASMR and ASDR of CVD attributable to lead exposure declined, whereas absolute deaths and DALYs remained substantial in 2021. Burden reductions were fastest in high-SDI settings, while several low-SDI regions showed slower declines or increasing trends (Tables 1, 2; Figures 1A–D). Globally, the mortality burden of CVD attributable to lead exposure showed a significant decline in ASMR, decreasing by −0.76% per year (95%CI: −0.85 to −0.67) from 1990 to 2021 (Table 1; Figures 1A,C). In 2021, the estimated number of deaths remained high at 1,476,238.28 (95%UI: −175,721.89 to 3,080,397.87; Table 1). Males experienced higher mortality counts than females in 2021 (847,993.59 [95%UI: −106,174.66 to 1,710,764.83] vs. 628,244.69 [95%UI: −69,547.23 to 1,331,127.94] deaths), while females exhibited a faster decline in ASMR (−0.86%/year [95%CI: −0.93 to −0.79] vs. −0.71%/year [95%CI: −0.81 to −0.61]; Table 1).

**Table 1:** Global and Regional analysis of CVD Attributable to Lead Exposure from 1990 to 2021: case counts (in hundreds), age-standardized mortality rates (per 100,000 population), and estimated annual percentage change (EAPC).

Location	Number(95%UI) 1990	ASMR(95%UI) 1990	Number(95%UI) 2021	ASMR(95%UI) 2021	EAPC(95%CI)
Global	796299.92(−90530.06,1670925.70)	22.37(−2.55,46.91)	1476238.28(−175721.89,3080397.87)	17.82(−2.11,37.19)	−0.76(−0.85,-0.67)
Sex					
Male	451133.59 (−51392.41, 916008.46)	29.02 (−3.31, 59.01)	847993.59 (−106174.66, 1710764.83)	23.49 (−2.92, 47.23)	−0.71 (−0.81, −0.61)
Female	345166.33 (−39137.64, 737663.80)	17.38 (−1.97, 37.09)	628244.69 (−69547.23, 1331127.94)	13.42 (−1.49, 28.44)	−0.86 (−0.93, −0.79)
SDI region					
High SDI	105853.00(−13883.16,226226.24)	9.63(−1.26,20.55)	109789.43(−12386.40,232697.60)	4.45(−0.50,9.42)	−2.60(−2.66,-2.53)
High-middle SDI	171723.93(−20064.81,368829.76)	19.90(−2.34,42.57)	298265.97(−35639.76,625651.23)	15.51(−1.84,32.46)	−0.91(−1.15,-0.67)
Middle SDI	263239.84(−26529.63,542230.47)	31.59(−3.14,64.81)	543366.47(−65285.55,1126880.07)	23.40(−2.79,48.36)	−0.97(−1.04,-0.89)
Low-middle SDI	180679.04(−21268.55,367977.60)	34.97(−4.01,70.93)	385799.05(−46415.02,785363.00)	31.43(−3.74,63.67)	−0.29(−0.37,-0.21)
Low SDI	73923.69(−7705.75,146641.74)	40.13(−4.09,78.99)	137751.11(−14456.01,270132.48)	35.10(−3.63,68.01)	−0.40(−0.51,-0.30)
GBD region					
Oceania	356.15(−35.64,746.67)	16.35(−1.63,34.32)	772.10(−75.45,1668.83)	13.90(−1.39,29.88)	−0.56(−0.64,-0.47)
Southeast Asia	50302.03(−5531.11,105613.34)	22.16(−2.44,46.66)	110879.08(−13275.60,234508.09)	19.43(−2.33,41.07)	−0.44(−0.61,-0.27)
East Asia	242033.76(−22869.34,508541.77)	37.24(−3.37,78.06)	487562.84(−58750.84,1022553.46)	25.75(−3.09,53.89)	−1.14(−1.31,-0.98)
Central Asia	8051.30(−1018.44,17401.20)	19.40(−2.45,41.86)	12401.80(−1501.81,26105.08)	19.19(−2.33,40.40)	−0.24(−0.61,0.14)
Eastern Europe	30549.92(−4119.60,67158.16)	12.26(−1.66,26.85)	38920.13(−4834.09,83975.44)	10.83(−1.35,23.38)	−0.95(−1.52,-0.37)
Central Europe	26430.80(−3388.83,55990.86)	19.51(−2.50,41.20)	29871.84(−3356.30,61531.62)	12.46(−1.40,25.66)	−1.68(−1.83,-1.53)
Australasia	3327.42(−460.46,7118.46)	14.76(−2.04,31.53)	2994.18(−394.94,6361.15)	4.71(−0.62,10.01)	−3.82(−3.91,-3.74)
High-income North America	33372.50(−4447.04,71750.77)	9.20(−1.22,19.76)	34602.68(−3639.82,73110.31)	4.80(−0.50,10.12)	−2.24(−2.35,-2.12)
High-income Asia Pacific	13248.88(−1600.66,28903.75)	7.42(−0.89,16.12)	15744.90(−1815.58,33401.66)	2.39(−0.28,5.12)	−3.64(−3.75,-3.53)
Western Europe	57691.03(−7697.29,122650.11)	9.70(−1.29,20.61)	54984.59(−5548.65,114296.33)	4.41(−0.46,9.21)	−2.57(−2.67,-2.47)
Southern Latin America	4358.12(−473.59,9278.26)	10.09(−1.09,21.46)	5013.17(−467.66,10654.62)	5.49(−0.52,11.66)	−1.76(−1.85,-1.66)
Andean Latin America	2554.58(−236.82,5296.77)	14.01(−1.28,29.01)	4839.59(−475.34,10125.64)	8.62(−0.84,18.03)	−1.61(−1.72,-1.50)
Caribbean	7440.30(−901.07,15256.73)	31.03(−3.73,63.47)	12676.51(−1334.48,25400.10)	23.21(−2.45,46.54)	−0.83(−0.89,-0.77)
Eastern Sub-Saharan Africa	24246.66(−2060.47,47718.12)	41.37(−3.32,81.83)	34442.84(−2978.94,67429.28)	27.80(−2.30,53.72)	−1.46(−1.54,-1.38)
Central Sub-Saharan Africa	5210.82(−446.97,10687.71)	30.12(−2.47,62.00)	12332.78(−1081.16,25386.53)	32.39(−2.68,66.07)	0.21(0.16,0.27)
Western Sub-Saharan Africa	19301.36(−1880.85,40664.13)	25.99(−2.54,54.59)	36212.76(−3809.84,71369.55)	23.56(−2.54,46.20)	−0.46(−0.63,-0.29)
Central Latin America	15690.94(−1795.11,31405.20)	22.61(−2.56,45.11)	35106.38(−4363.51,72073.84)	14.94(−1.86,30.61)	−1.53(−1.66,-1.40)
Tropical Latin America	18926.83(−2208.93,39244.31)	24.03(−2.78,49.68)	26534.40(−2961.35,54309.95)	10.74(−1.19,21.99)	−2.43(−2.53,-2.33)
North Africa and Middle East	80366.00(−8664.14,162063.01)	59.19(−6.22,119.07)	143158.47(−15112.26,289024.09)	40.09(−4.18,80.50)	−1.26(−1.30,-1.22)
South Asia	149240.76(−18763.79,304184.57)	30.61(−3.74,61.94)	368557.66(−46484.00,748649.84)	29.21(−3.63,58.70)	−0.07(−0.18,0.04)
Southern Sub-Saharan Africa	3599.77(−308.87,7276.73)	15.10(−1.27,30.76)	8629.59(−763.03,17326.14)	18.42(−1.61,36.31)	0.77(0.27,1.27)

**Table 2:** Global and regional analysis of CVD burden attributable to lead exposure (1990–2021): case counts (in hundreds), age-standardized DALY rates (per 100,000 population), and estimated annual percentage change (EAPC).

Location	Number 1990	ASDR 1990	Number 2021	ASDR 2021	EAPC(95% CI)
Global	19030685.01(−2151614.92,39936969.57)	484.95(−54.93,1016.51)	30017542.38(−3660038.64,62164622.01)	351.36(−42.73,727.75)	−1.09(−1.18,-0.99)
Sex					
Male	11410724.45(−1297965.37,23203751.29)	629.58(−71.66,1280.87)	18180436.51(−2315559.50,36956474.37)	463.24(−58.70,938.78)	−1.03(−1.13,-0.92)
Female	7619960.56(−853649.55,16361337.41)	363.00(−40.74,778.53)	11837105.86(−1327537.03,24806835.69)	255.67(−28.63,535.84)	−1.19(−1.28,-1.11)
SDI Region					
High SDI	2070533.16(−274155.58,4440734.45)	189.22(−24.97,405.48)	1837668.74(−210608.74,3851037.92)	84.54(−9.58,177.29)	−2.68(−2.73,-2.63)
High-middle SDI	3839561.19(−442547.34,8229713.99)	401.25(−46.42,858.16)	5284016.72(−654346.71,11150351.72)	269.70(−33.27,569.36)	−1.45(−1.71,-1.20)
Middle SDI	6489877.84(−661568.99,13453855.11)	651.09(−65.84,1340.01)	10875417.24(−1315599.29,22606994.92)	426.18(−51.41,885.22)	−1.38(−1.46,-1.31)
Low-middle SDI	4693821.98(−559496.50,9631882.08)	769.85(−90.67,1569.13)	8742161.56(−1057590.37,17882167.28)	630.82(−76.03,1287.66)	−0.60(−0.69,-0.52)
Low SDI	1917323.75(−201229.61,3811213.41)	866.89(−90.17,1718.09)	3253702.36(−342025.75,6439620.91)	687.52(−72.23,1352.17)	−0.79(−0.88,-0.71)
GBD Region					
Southeast Asia	1389350.00(−152181.97,2918671.34)	522.38(−57.39,1095.61)	2651827.70(−313518.40,5583175.62)	409.91(−48.58,864.92)	−0.80(−0.97,-0.63)
Oceania	9647.69(−941.96,20451.59)	342.50(−34.04,718.91)	19553.83(−1859.11,42332.92)	278.66(−27.23,602.94)	−0.70(−0.78,-0.61)
East Asia	5836503.61(−567392.67,12217553.18)	727.37(−68.76,1520.87)	9015754.46(−1074653.41,18928423.08)	436.63(−51.93,917.19)	−1.62(−1.77,-1.48)
Eastern Europe	643698.40(−86667.15,1418714.44)	240.19(−32.38,528.57)	714396.65(−88475.07,1531600.03)	202.80(−25.10,433.75)	−1.18(−1.82,-0.54)
Central Asia	177989.69(−22829.08,384825.44)	392.29(−50.21,849.06)	251838.40(−30755.80,531273.27)	342.25(−41.92,722.69)	−0.76(−1.19,-0.34)
Central Europe	555666.77(−72347.56,1183637.59)	384.35(−49.94,817.62)	488888.75(−56115.03,1009092.08)	213.10(−24.44,440.42)	−2.18(−2.34,-2.01)
Australasia	63377.37(−8871.83,135398.73)	273.73(−38.26,584.30)	45360.74(−6197.59,96161.24)	78.04(−10.62,165.28)	−4.18(−4.24,-4.11)
High-income Asia Pacific	281273.95(−33758.96,611213.39)	145.04(−17.33,314.72)	237755.86(−28045.76,506958.09)	44.73(−5.37,96.30)	−3.84(−3.91,-3.78)
Caribbean	167453.07(−20104.33,341551.92)	652.70(−78.51,1331.50)	255891.72(−27108.90,512774.91)	473.95(−50.22,950.09)	−0.93(−0.99,-0.87)
Western Europe	1036834.53(−141139.78,2213236.63)	178.98(−24.32,381.66)	759325.04(−83561.21,1589352.06)	69.42(−7.93,145.22)	−3.13(−3.23,-3.03)
Southern Latin America	97178.90(−10989.44,207673.02)	213.56(−24.06,456.43)	89487.46(−9054.57,190587.41)	101.10(−10.27,215.15)	−2.32(−2.40,-2.24)
High-income North America	633627.93(−83484.65,1349766.32)	181.18(−23.64,385.04)	604673.61(−61516.07,1251086.61)	91.10(−8.98,187.89)	−2.27(−2.36,-2.19)
Tropical Latin America	470746.37(−54842.84,981451.10)	514.22(−60.16,1068.88)	539857.16(−62134.36,1112873.10)	211.99(−24.30,436.87)	−2.79(−2.89,-2.69)
Andean Latin America	57188.81(−5487.54,119071.74)	283.08(−26.83,586.09)	94904.26(−9585.24,200312.56)	163.20(−16.45,344.12)	−1.83(−1.95,-1.71)
Central Latin America	343327.69(−40667.85,693803.54)	432.47(−50.81,870.97)	652208.78(−82805.96,1353926.24)	267.35(−33.95,554.08)	−1.79(−1.91,-1.67)
North Africa and Middle East	1935307.26(−214631.76,3906025.52)	1192.72(−130.11,2405.71)	3034849.80(−325405.81,6195160.38)	725.10(−77.44,1468.23)	−1.65(−1.71,-1.59)
South Asia	3996129.77(−508297.13,8249067.02)	688.21(−86.29,1404.18)	8378456.03(−1067051.70,17223636.81)	587.60(−74.41,1201.49)	−0.46(−0.55,-0.38)
Southern Sub-Saharan Africa	93551.24(−8119.39,191284.99)	337.45(−29.37,687.58)	201049.24(−17937.11,409679.11)	364.72(−32.56,734.80)	0.36(−0.14,0.87)
Eastern Sub-Saharan Africa	614863.16(−52997.24,1218616.19)	863.23(−72.72,1705.60)	803699.17(−71076.23,1593914.34)	527.58(−45.86,1034.04)	−1.80(−1.89,-1.71)
Central Sub-Saharan Africa	140419.93(−12235.58,289167.12)	645.05(−55.13,1329.45)	301884.06(−27445.40,619283.03)	620.22(−54.41,1264.16)	−0.17(−0.23,-0.10)
Western Sub-Saharan Africa	486548.87(−46328.73,1021872.94)	560.41(−54.04,1180.55)	875879.66(−88890.58,1733268.72)	469.39(−49.27,923.99)	−0.71(−0.90,-0.53)

Marked SDI disparities were observed. High-SDI regions experienced a sharp decline in ASMR (−2.60%/year [95%CI: −2.66 to −2.53]) with relatively stable death counts (105,853.00 [95%UI: −13,883.16 to 226,226.24] in 1990 and 109,789.43 [95%UI: −12,386.40 to 232,697.60] in 2021), whereas low-SDI regions showed an 86.3% increase in deaths (73,923.69 [95%UI: −7,705.75 to 146,641.74] in 1990 and 137,751.11 [95%UI: −14,456.01 to 270,132.48] in 2021) and the slowest ASMR decline (−0.40%/year [95%CI: −0.51 to −0.30]; Table 1; Figures 1A,C). Regionally, Australasia (−3.82%/year [95%CI: −3.91 to −3.74]) and Western Europe (−2.57%/year [95%CI: −2.67 to −2.47]) achieved the most substantial ASMR reductions, while Southern Sub-Saharan Africa (+0.77%/year [95%CI: 0.27 to 1.27]) and Central Africa (+0.21%/year [95%CI: 0.16 to 0.27]) showed increasing trends (Table 1; Figures 1A,C). East Asia exhibited a decline in ASMR (−1.14%/year [95%CI: −1.31 to −0.98]) but a 101% increase in absolute deaths (242,033.76 [95%UI: −22,869.34 to 508,541.77] in 1990 and 487,562.84 [95%UI: −58,750.84 to 1,022,553.46] in 2021; Table 1; Figures 1A,C), illustrating divergence between declining age-standardized rates and rising counts.

Globally, the DALYs burden remained substantial but declined in ASDR from 1990 to 2021, with an annual decrease of 1.09% (95%CI: −1.18 to −0.99; Table 2; Figures 1B,D). In 2021, DALYs reached 30,017,542.38 (95%UI: −3,660,038.64 to 62,164,622.01), with a 95% uncertainty interval that included a negative lower bound (Table 2). Males had higher DALY counts than females in 2021 (18,180,436.51 [95%UI: −2,315,559.50 to 36,956,474.37] vs. 11,837,105.86 [95%UI: −1,327,537.03 to 24,806,835.69] DALYs), while females exhibited a faster decline in ASDR (−1.19%/year [95%CI: −1.28 to −1.11] vs. −1.03%/year [95%CI: −1.13 to −0.92]; Table 2).

SDI-stratified analyses showed pronounced inequalities. High-SDI regions exhibited rapid declines in ASDR (−2.68%/year [95%CI: −2.73 to −2.63]), whereas low-SDI regions experienced a 70% increase in absolute DALYs (1,917,323.75 [95%UI: −201,229.61 to 3,811,213.41] in 1990 vs. 3,253,702.36 [95%UI: −342,025.75 to 6,439,620.91] in 2021) with the slowest ASDR decline (−0.79%/year [95%CI: −0.88 to −0.71]; Table 2; Figures 1B,D). Regional heterogeneity was also evident: Australasia (−4.18%/year [95%CI: −4.24 to −4.11]) and Western Europe (−3.13%/year [95%CI: −3.23 to −3.03]) achieved the fastest declines, whereas Southern Sub-Saharan Africa showed an increase in ASDR (+0.36%/year [95%CI: −0.14 to 0.87]; Table 2; Figures 1B,D). East Asia showed declining ASDR (−1.62%/year [95%CI: −1.77 to −1.48]) but increasing absolute DALYs (5,836,503.61 [95%UI: −567,392.67 to 12,217,553.18] in 1990 vs. 9,015,754.46 [95%UI: −1,074,653.41 to 18,928,423.08] in 2021; Table 2; Figures 1B,D).

### 3.2 Age- and sex-stratified analysis

In 2021, global cardiovascular disease (CVD) mortality attributable to lead exposure showed clear age- and sex-specific disparities, with the burden increasing steadily with age and consistently higher in men than in women. Deaths were concentrated in older age groups, particularly among individuals aged 85–89 years, with approximately 106,000 deaths in men (mortality rate: 614.7 per 100,000) and 96,000 in women (335.6 per 100,000), yielding a male-to-female mortality rate ratio of about 1.83. Mortality rates remained high in those aged ≥95 years (865.4 per 100,000 in men and 714.1 per 100,000 in women). Substantial mortality was also observed in middle-aged adults; for example, in the 55–59-year group, there were about 55,000 deaths in men (28.2 per 100,000) and 28,000 in women (14.2 per 100,000; Supplementary Figure 1A; Supplementary Table 1).

A similar pattern was evident for disability-adjusted life years (DALYs), with rates rising sharply with age and peaking in the oldest groups (7,297.8 per 100,000 in men and 5,965.4 per 100,000 in women aged ≥95 years). Individuals aged 85–89 years also experienced a heavy DALYs burden, with rates of 6,337.4 per 100,000 in men and 3,489.4 per 100,000 in women. In absolute terms, the largest DALYs contribution came from those aged 55–64 years, particularly the 60–64-year group, with approximately 2.246 million DALYs in men (1,444.1 per 100,000) and 1.218 million in women (740.5 per 100,000). Even in younger adults aged 25–44 years, DALYs rates remained higher in men than in women (e.g., 56.0 vs. 25.9 per 100,000 at ages 30–34). Overall, older men represent the population at highest risk for lead exposure–related CVD mortality and disease burden (Supplementary Figure 1B; Supplementary Table 1).

### 3.3 Decomposition analysis results

**Figure 2:**
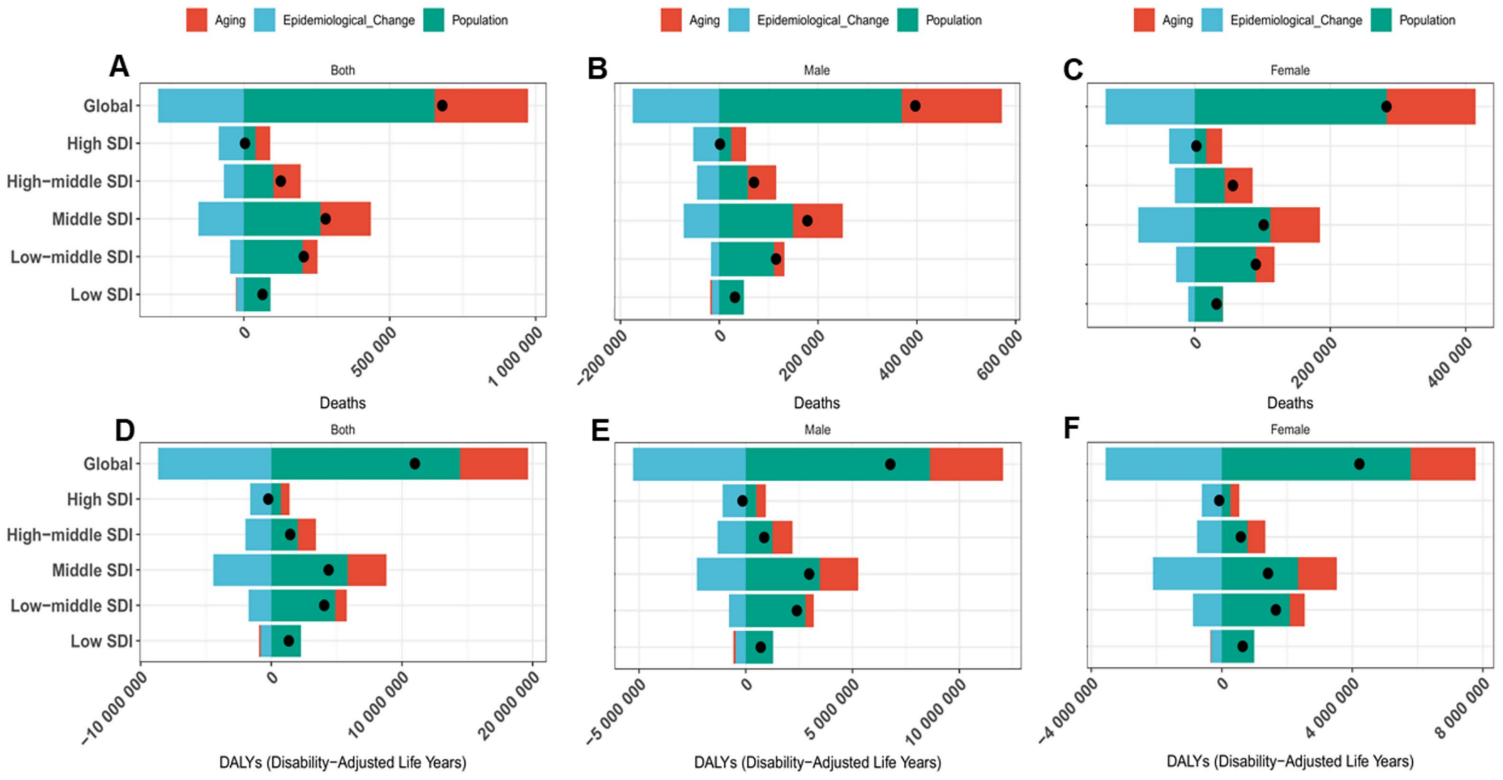
Decomposition analysis of CVD attributable to lead exposure mortality and disability-adjusted life years (DALYs) burden. **(A–C)** ASMR. **(D–F)** ASDR.

PAD decomposition indicated that changes in global lead exposure-attributable CVD burden from 1990 to 2021 were driven by population growth, population aging, and epidemiological change (Figure 2; Supplementary Table 2). Population growth was the leading contributor, accounting for 96.07% (+653,189 deaths) of the increase in global mortality (Figure 2A; Supplementary Table 2) and 131.44% of DALYs growth (Figure 2D; Supplementary Table 2). This contribution was particularly pronounced in low-SDI regions (143.15% for mortality and 170.43% for DALYs; Figures 2A,D; Supplementary Table 2). Population aging contributed 47.12% (+320,385 deaths) to mortality change and 47.39% to DALY change, with the largest aging impact observed in middle-SDI regions (+61.83% for mortality and +67.71% for DALYs; Figures 2A,D; Supplementary Table 2). Epidemiological change reduced global mortality by 43.19% (−293,635 deaths) and DALYs by 78.83% (Figures 2A,D; Supplementary Table 2). Sex-stratified decomposition patterns are presented in Figures 2B,C,E,F (Supplementary Table 2).

### 3.4 Health inequality analysis

**Figure 3:**
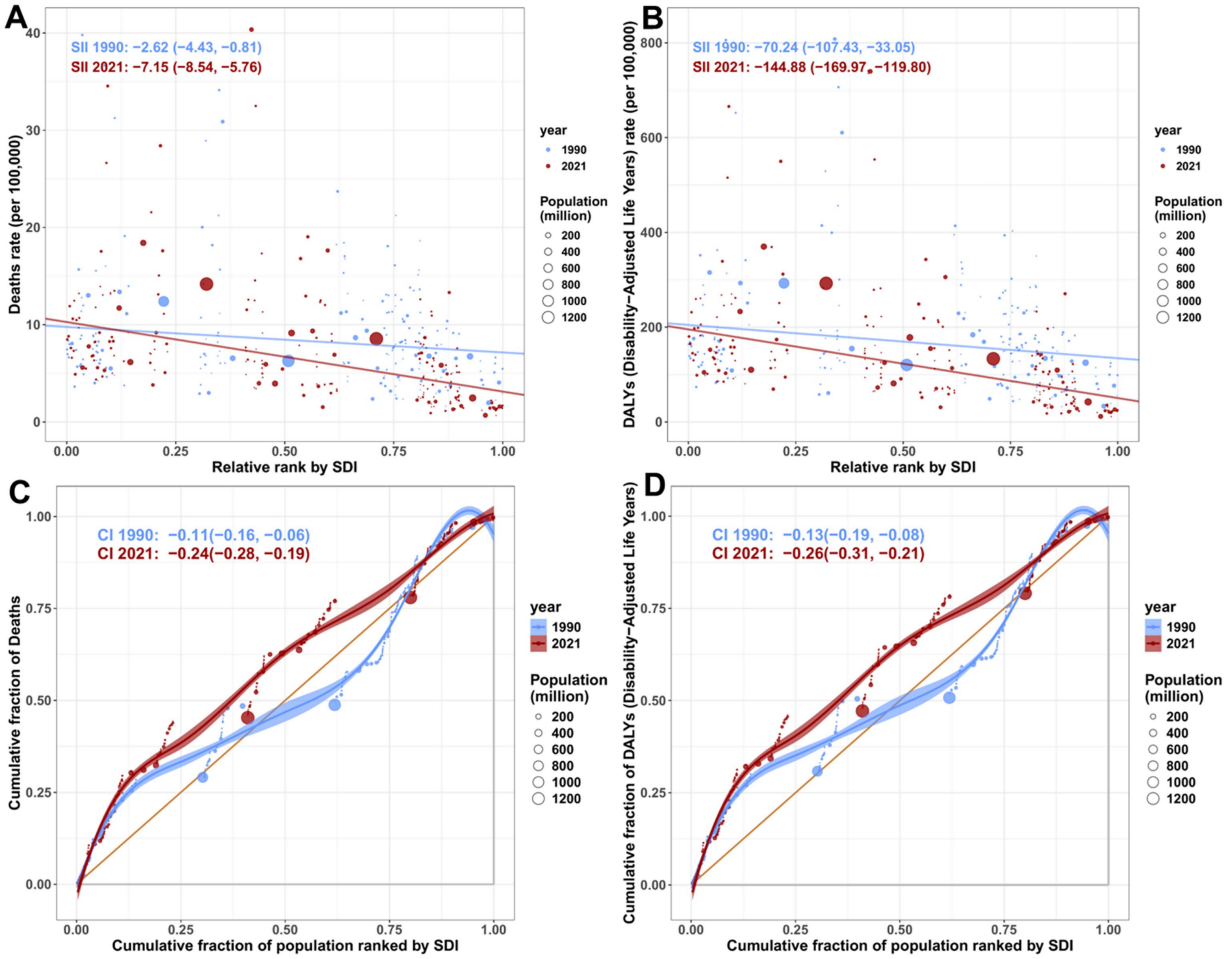
Regression and concentration curves of global health inequalities in deaths **(A,C)** and disability-adjusted life years (DALYs) **(B,D)** of CVD attributable to lead exposure in 1990 and 2021. Panels A and B show the slope index of inequality, depicting the relationship between the SDI and deaths and DALYs, where each point represents a country or region, and the size of the point is determined by population size. Panels C and D show the concentration index, which quantifies relative inequality by integrating the area under the Lorenz curve and aligning the distribution of deaths and DALYs with the population distribution by SDI. Blue represents the data for 1990, and red represents the data for 2021. DALYs, disability-adjusted life years; SDI, social deprivation index.

We assessed socioeconomic inequality in lead exposure-attributable CVD burden using the slope index of inequality (SII) and concentration curves with the concentration index (CI; Figure 3). Both metrics indicated that mortality and DALY burdens were disproportionately concentrated in lower-SDI populations, and inequality increased from 1990 to 2021. For ASMR, the SII decreased from −2.62 (95%CI: −4.43 to −0.81) in 1990 to −7.15 (−8.54 to −5.76) in 2021 (Figure 3A), indicating a widening absolute gap across the SDI distribution. The concentration curves were further from the line of equality in 2021 than in 1990 (Figure 3C), and the CI became more negative, shifting from −0.11 (−0.16 to −0.06) in 1990 to −0.24 (−0.28 to −0.19) in 2021. For ASDR, inequality was more pronounced and similarly worsened. The SII declined from −70.24 (−107.43 to −33.05) in 1990 to −144.88 (−169.97 to −119.80) in 2021 (Figure 3B). Consistently, the CI for DALYs decreased from −0.13 (−0.19 to −0.08) in 1990 to −0.26 (−0.31 to −0.21) in 2021 (Figure 3D),v indicating increasing concentration of DALY losses among lower-SDI populations.

### 3.5 Frontier analysis

**Figure 4:**
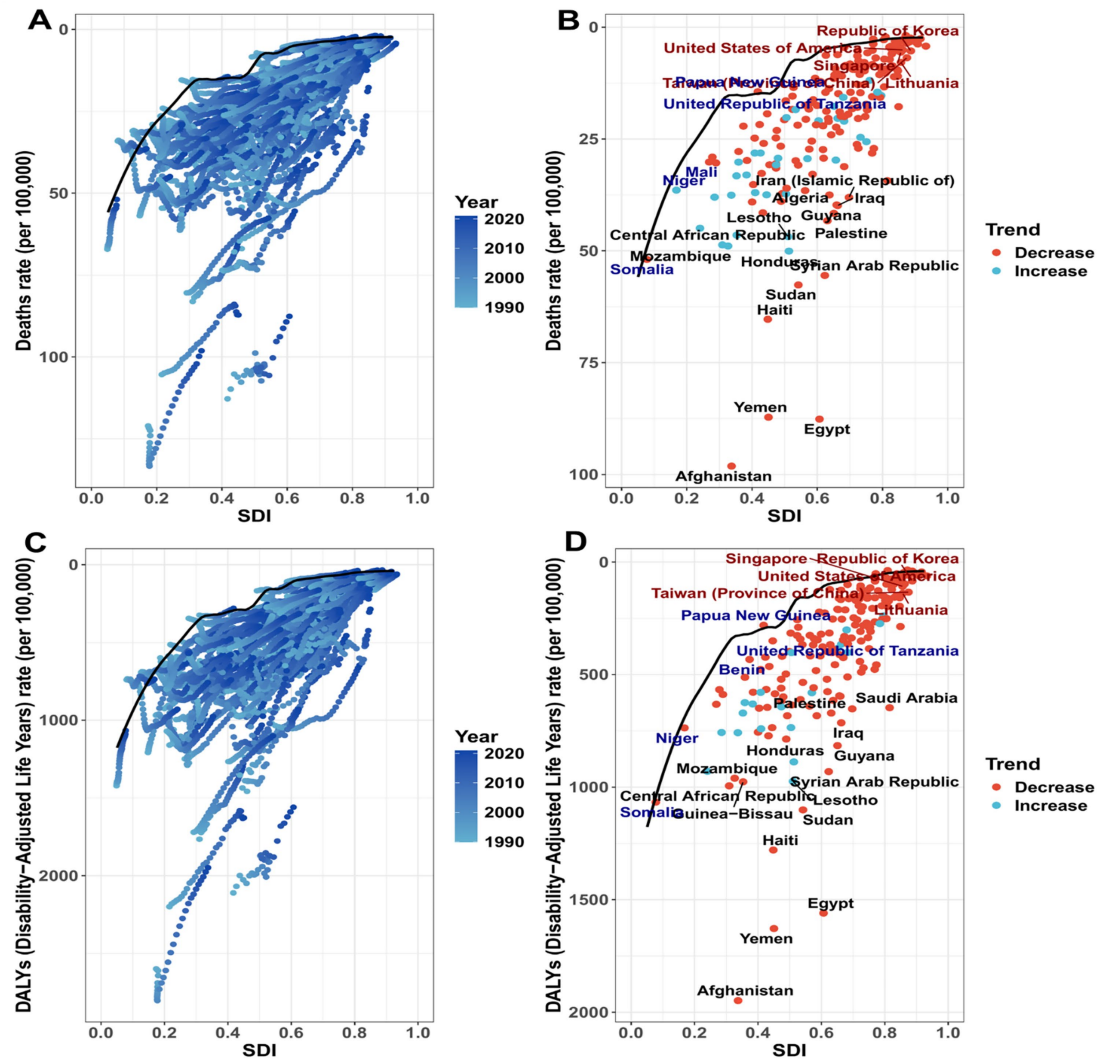
The relationship between the Social Development Index (SDI) from 1990 to 2021 and the ASMR and ASDR of CVD attributable to lead exposure in 204 countries and regions. In figures **(A,C)**, the color gradually changes from light green (1990) to dark green (2021), indicating the passage of time. In figures **(B,D)**, each point represents a country or region in 2021; the black solid line is the frontier boundary; the 15 countries/regions with the largest gap from the frontier are highlighted in brown. The blue points represent those with low SDI and the smallest gap from the frontier; the red points represent those with high SDI and the largest gap from the frontier. The color of the points also reflects the direction of change in ASR from 1990 to 2021: orange indicates a decrease, and green indicates an increase.

Frontier analysis based on GBD 2021 demonstrated substantial cross-country heterogeneity in prevention potential at comparable SDI levels for both mortality and DALYs (Figures 4A–D; Supplementary Tables 3, 4). For mortality, high-SDI countries such as Switzerland and Norway showed low observed rates and small efficiency differences relative to the frontier, whereas several low-SDI countries (e.g., Afghanistan and Yemen) had high observed rates and large efficiency differences (Figures 4A,B; Supplementary Tables 3, 4). Countries with similar SDI (e.g., Cambodia and Laos) displayed notable differences in observed mortality burden and efficiency differences (Figures 4A,B; Supplementary Tables 3, 4). For DALYs, high-burden countries were concentrated in low-SDI settings, with large efficiency differences observed in Afghanistan, Yemen, and Egypt (Figures 4C,D; Supplementary Tables 3, 4). Some middle-SDI countries (e.g., Iraq and Palestine) also exhibited large efficiency differences (Figures 4C,D; Supplementary Tables 3, 4). Special cases showing an efficiency difference of 0 (e.g., Somalia and Papua New Guinea) are reported in Supplementary Tables 3, 4 for verification and sensitivity assessment (Figures 4C,D; Supplementary Tables 3, 4).

### 3.6 Subtype-specific burden analysis of CVD

**Figure 5:**
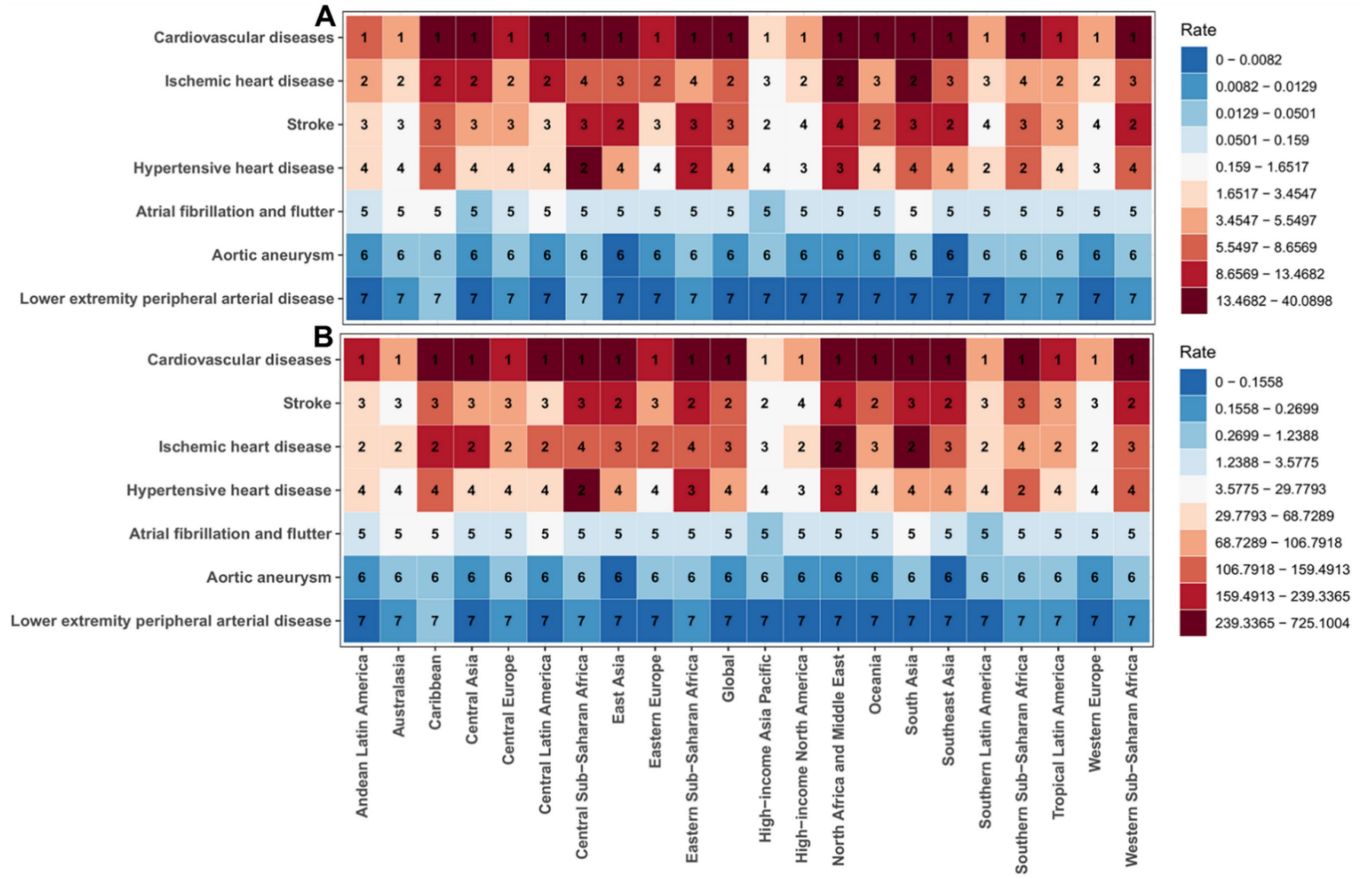
Age-standardized mortality rates (ASMR) and age-standardized death risks (ASDR) of CVD subtypes attributable to lead exposure across global regions. **(A)** Geographic distribution of CVD ASMR, with color intensity representing mortality magnitude (darker shades indicate higher ASMR values). **(B)** Corresponding geographic distribution of CVD ASDR, using identical color scaling to represent risk gradients.

Subtype analyses for 2021 showed pronounced regional heterogeneity in lead exposure-attributable CVD mortality and DALYs (Figures 5A,B). For ASMR, ischemic heart disease and stroke were the dominant contributors globally (7.48 and 7.05 per 100,000, respectively), with particularly high rates in East Asia (IHD 10.47 per 100,000; stroke 15.86 per 100,000; Figure 5A). Eastern Europe and the Caribbean had high IHD mortality (11.59 and 11.37 per 100,000, respectively), while the Caribbean showed higher hypertensive heart disease mortality (6.83 per 100,000; Figure 5A). Western Europe had the highest AF/AFL mortality (0.29 per 100,000), and aortic aneurysm mortality exhibited higher values in high-income Australasia and the Caribbean (Figure 5A; Supplementary Table 5).

For ASDR, stroke and IHD remained the leading contributors globally (152.36 and 150.22 per 100,000, respectively; Figure 5B). East Asia had the highest stroke DALYs (319.72 per 100,000), while the Caribbean showed high IHD ASDR (225.12 per 100,000; Figure 5B). Hypertensive heart disease DALYs were notable in sub-Saharan Africa (e.g., Central Africa 83.57 per 100,000) and Southeast Asia (73.24 per 100,000; Figure 5B; Supplementary Table 5).

### 3.7 ARIMA analysis

**Figure 6:**
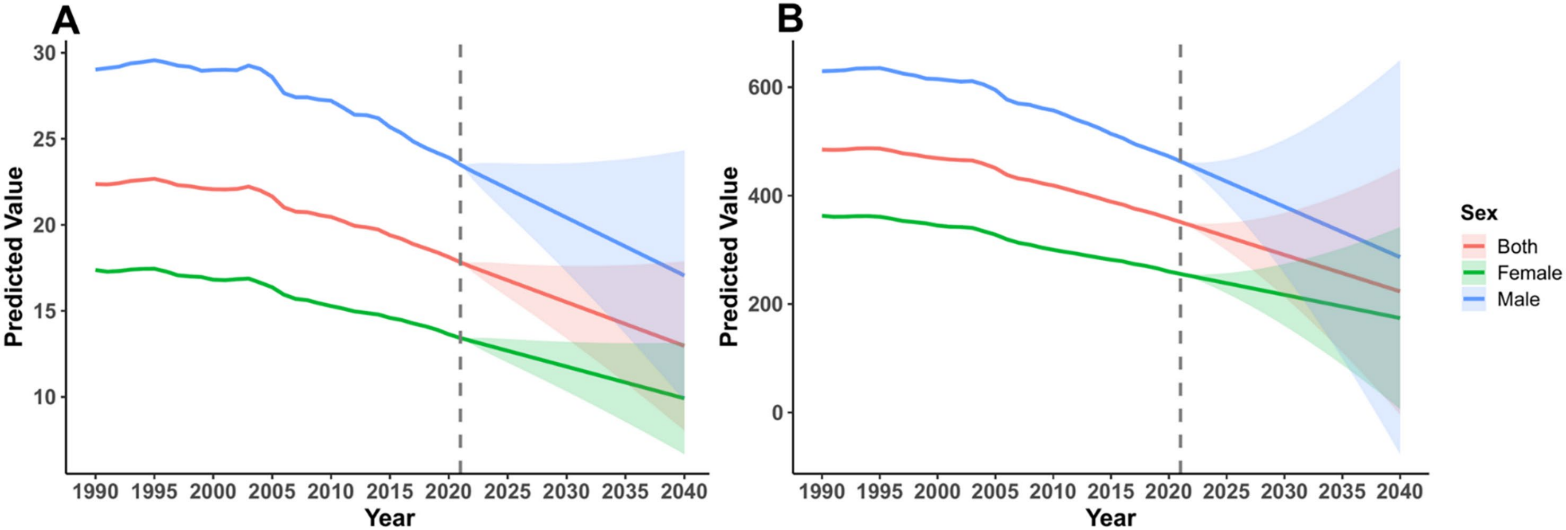
ARIMA model-based projections of age-standardized mortality rates (ASMR) and age-standardized DALYs rate (ASDR) for CVD attributable to lead exposure (1990–2040). **(A)** Temporal trends in ASMR stratified by sex (overall, male, female populations). **(B)** Corresponding temporal trends in ASDR with identical sex stratification.

ARIMA projections indicated continued declines in ASMR through 2040 for males, females, and both sexes combined (Figure 6A; Supplementary Tables 6, 7). For mortality, the ASMR declined from 29.02 per 100,000 (males) and 17.38 per 100,000 (females) in 1990 to projected 17.06 per 100,000 and 9.92 per 100,000 in 2040, respectively; the overall rate declined from 22.37 to 12.96 per 100,000 (Figure 6A; Supplementary Tables 6, 7). For ASDR, males decreased from 629.58 per 100,000 in 1990 to 463.24 per 100,000 in 2021, while females decreased from 362.99 to 255.67 per 100,000; projections suggested further declines by 2040 (Figure 6B; Supplementary Tables 6, 7).

To align with Frontiers reporting style and improve readability, we present the projected trajectories and key endpoints in the main text (Figures 6A,B) and refer detailed model diagnostics (e.g., KPSS statistics, differencing decisions, Ljung–Box tests, and ARIMA orders) to Supplementary Tables 6, 7 (Figures 6A,B; Supplementary Tables 6, 7). Prediction intervals widened with increasing forecast horizon, reflecting greater uncertainty in long-term projections (Figures 6A,B; Supplementary Tables 6, 7).

All estimates are presented with 95% uncertainty intervals (UIs), calculated as the 2.5th and 97.5th percentiles of the posterior draw distribution (GBD framework). Wider UIs may occur in regions or outcomes with sparse or heterogeneous input data and should be interpreted with caution. ASMR: Age-Standardized Mortality Rates;

CVD: cardiovascular disease.

All estimates are presented with 95% uncertainty intervals (UIs), calculated as the 2.5th and 97.5th percentiles of the posterior draw distribution (GBD framework). Wider UIs may occur in regions or outcomes with sparse or heterogeneous input data and should be interpreted with caution. ASDR: Age-standardized DALY rates; CVD: cardiovascular disease.

## 4 Discussion

Based on estimates from the Global Burden of Disease (GBD) 2021 Study covering the period 1990–2021, this study yielded four key findings. First, globally, the age-standardized mortality rate (ASMR) and disability-adjusted life year (DALY) rate of cardiovascular diseases (CVDs) attributable to lead exposure have shown a downward trend. However, the absolute number of deaths and DALYs in 2021 remained high, indicating that demographic pressures continue to offset the positive impacts of prevention and control efforts. Second, socioeconomic inequality has been pronounced and is worsening over time. Both the Slope Index of Inequality (SII) and the Concentration Index analyses demonstrated that the burden of lead exposure-attributable CVD is increasingly concentrated in regions with lower sociodemographic index (SDI) levels. Third, decomposition analysis revealed that population growth and aging were the major contributors to the increase in absolute disease burden, while epidemiological changes partially offset the effects of these demographic factors. Fourth, frontier analysis indicated substantial efficiency gaps in disease control among countries with similar SDI levels, suggesting that considerable potential remains for reducing the burden of lead exposure-attributable CVD—particularly in low- and middle-SDI countries.

The observed global decline in ASMR and ASDR for lead exposure-attributable CVD suggests that certain regions have achieved progress in CVD prevention and lead exposure control. However, the persistently high absolute burden underscores that population growth and aging remain strong counterbalancing forces. This trend aligns with a common conclusion from previous GBD studies—namely, that improvements in age-specific rates can coexist with rising absolute disease counts when population structures change rapidly (25, 26). The decomposition analysis in the present study provides quantitative evidence for this phenomenon: population growth and aging contributed positively to the overall disease burden, whereas epidemiological transitions exerted a mitigating effect.

This study found that ischemic heart disease (IHD) accounted for the largest proportion of lead exposure-attributable CVD burden globally, whereas stroke was the leading condition in specific regions. These compositional differences may reflect variations in baseline CVD characteristics, competing risk factors, and temporal shifts in the distribution of risk factors across regions (27, 28). The substantial contribution of hypertensive heart disease highlights the importance of blood pressure–related pathophysiological pathways as potential clinical targets for the prevention of lead-associated CVD. It should be emphasized that these compositional patterns represent lead-attributable fractions estimated under the GBD comparative risk assessment framework, rather than clinical diagnoses directly caused by lead exposure at the individual level.

Inequality analysis indicated that from 1990 to 2021, both ASMR and ASDR burdens of lead exposure-attributable CVD became increasingly concentrated among populations with lower SDI levels, with inequality in DALYs being more pronounced than that in mortality. This pattern suggests that beyond premature death, disability and quality-of-life loss attributable to lead exposure are disproportionately borne by populations in socioeconomically disadvantaged regions. The persistence and widening of such health inequalities likely result from the combined effects of multiple structural factors, including disparities in environmental regulation, industrial production standards, housing and consumer safety, occupational protection, and access to CVD prevention and treatment services (29–31). However, as GBD estimates are based on ecological data, causal relationships between these inequalities and specific factors cannot be inferred.

Frontier analysis provides an intuitive benchmark for assessing disease burden by quantifying the gap between a country’s observed burden and the lowest burden achieved by other countries with comparable SDI levels (32, 33). The substantial efficiency gaps observed in many low- and some middle-SDI countries suggest that substantial prevention potential exists even without immediate improvements in SDI. These findings should be interpreted as evidence-based signals, indicating that through enhanced environmental governance, improved monitoring systems, and strengthened healthcare service capacity, countries at similar development levels could achieve much lower CVD burdens attributable to lead exposure.

The findings of this study have several practical implications. First, source control remains fundamental: phasing out existing lead sources—including industrial emissions, informal lead recycling, lead-containing products, and paints—and strengthening regulatory enforcement can yield extensive health benefits. Second, integrating lead exposure control into CVD prevention systems is critical in high-burden regions. In areas with sufficient capacity, blood lead monitoring can be combined with hypertension screening, CVD risk stratification, and access to essential cardiovascular medications to target the primary downstream pathways of disease burden. Third, SDI-stratified prevention strategies can improve intervention efficiency. High-SDI regions should focus on maintaining low exposure levels through continuous monitoring and regulation; middle-SDI regions could benefit from targeted control of industrial and consumer lead content and the expansion of community-based CVD prevention programs; low-SDI regions should prioritize developing basic surveillance capacity, reducing exposure in high-risk areas, and implementing cost-effective CVD prevention strategies. Finally, since the disease burden is concentrated in socioeconomically disadvantaged regions, the global community can help close the substantial efficiency gap identified in the frontier analysis through technology transfer, laboratory capacity building, and implementation funding support.

The autoregressive integrated moving average (ARIMA) model in this study projected that the age-standardized rates of lead exposure-attributable CVD will continue to decline between 2021 and 2040. However, the widening prediction intervals over time indicate increasing uncertainty in long-term projections. Therefore, these projections should be interpreted as scenario-based extrapolations rather than deterministic forecasts. Future trends in disease burden will depend on the intensity of lead source control, regulatory enforcement, healthcare accessibility, and broader environmental and economic transitions.

### 4.1 Limitations

This study has several limitations. First, part of the GBD estimation process relies on model extrapolation, particularly in data-scarce regions, which may result in wider uncertainty intervals. The conclusions also depend on assumptions about lead exposure distribution and the shape of risk functions (34). Second, the results of the comparative risk assessment depend on counterfactual exposure assumptions and the generalizability of relative risk estimates, and this approach may not fully capture heterogeneity across populations (35). Third, as an ecological analysis, this study cannot establish causal relationships at the individual level. Residual confounding may persist due to multiple exposures (e.g., air pollution) and interrelated social determinants of health. Fourth, although frontier and decomposition analyses are useful for benchmarking and identifying key drivers, they cannot directly reveal causal mechanisms (36). Therefore, the results should be interpreted as population-level signals that inform prevention priority setting and hypothesis generation rather than causal inference.

## 5 Conclusion

In summary, lead exposure-attributable CVD burden shows declining age-standardized rates but persistently high absolute counts, widening inequality concentrated in lower-SDI settings, and large cross-country efficiency gaps. These patterns indicate that progress in exposure reduction and cardiovascular prevention has been uneven, underscoring the need for SDI-tailored policies that combine source control with scalable CVD prevention and strengthened monitoring, especially in LMICs.

## Data Availability

The original contributions presented in the study are included in the article/Supplementary material, further inquiries can be directed to the corresponding author.

## Footnotes

1
https://vizhub.healthdata.org/gbd-results/

## Glossary

CVD: Cardiovascular Disease
GBD: Global Burden of Disease
DALYs: Disability-Adjusted Life Years
ASMR: Age-Standardized Mortality Rate
ASDR: Age-Standardized DALY Rate
SDI: Socio-demographic Index
SII: Slope Index of Inequality
CI: Concentration Index
ARIMA: Autoregressive Integrated Moving Average
EAPC: Estimated Annual Percentage Change
YLLs: Years of Life Lost
YLDs: Years Lived with Disability
UI: Uncertainty Interval
LMICs: Low- and Middle-Income Countries
IHD: Ischemic Heart Disease
AF/AFL: Atrial Fibrillation and Flutter
LEPAD: Lower Extremity Peripheral Arterial Disease
PAD: Peripheral Arterial Disease
PAF: Population Attributable Fraction
RLM: Robust Linear Regression
LOESS: Locally Weighted Scatterplot Smoothing
AIC: Akaike Information Criterion
BIC: Bayesian Information Criterion
ADF: Augmented Dickey–Fuller
KPSS: Kwiatkowski–Phillips–Schmidt–Shin
GHDx: Global Health Data Exchange
CODEm: Cause of Death Ensemble model
