# Supplementary material for: Global burden of cardiovascular disease attributable to lead exposure: based on the global burden of disease study 2021
Source: Front Public Health. 2026 Feb 27;14:1690287. doi: 10.3389/fpubh.2026.1690287 (PMC12982341; doi:10.3389/fpubh.2026.1690287)
Supplement: Supplementary file 1 [file Data_Sheet_1.docx]

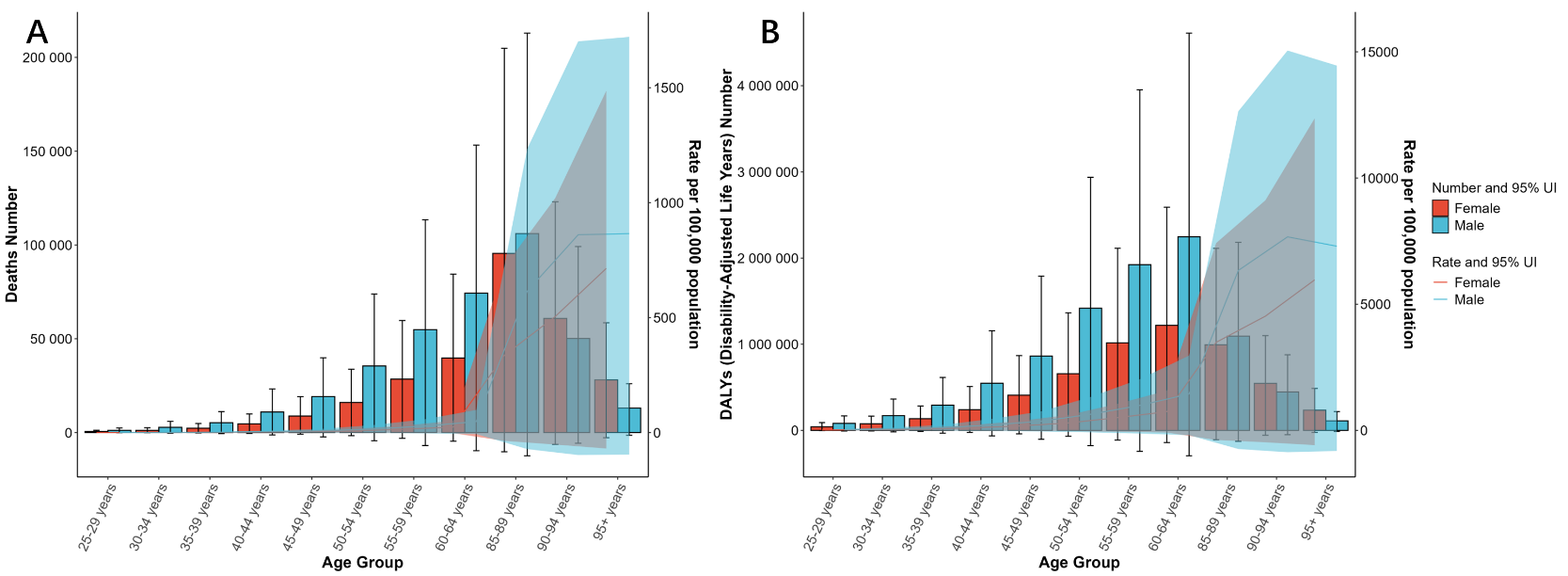


Supplementary Figure 1. Age- and sex-stratified analysis of the burden of cardiovascular disease attributable to lead exposure in 2021.

Supplementary Table 1: Global burden of cardiovascular disease attributable to lead exposure in 2021, by sex and age group: Deaths and DALYs with 95% uncertainty intervals.

| **Valuable** | **Sex** | **Age** | **Numbers(95%UI)** | **ASR(95%UI)** |
| --- | --- | --- | --- | --- |
| Deaths | Male | 85-89 years | 106058.89(-12385.22，212941.21) | 614.74(-71.79，1234.25) |
| Deaths | Female | 85-89 years | 95537.23(-10235.09，204859.09) | 335.58(-35.95，719.58) |
| Deaths | Male | 90-94 years | 50154.34(-5684.47，99178.92) | 860.50(-97.53，1701.62) |
| Deaths | Female | 90-94 years | 60907.30(-6270.91，123022.33) | 505.00(-51.99，1020.01) |
| Deaths | Male | 95+ years | 13085.00(-1444.20，26024.01) | 865.39(-95.51，1721.12) |
| Deaths | Female | 95+ years | 28123.09(-2722.98，58534.80) | 714.10(-69.14，1486.31) |
| Deaths | Male | 25-29 years | 1207.14(-105.75，2481.07) | 0.41(-0.04，0.83) |
| Deaths | Female | 25-29 years | 596.06(-40.82，1252.16) | 0.20(-0.01，0.43) |
| Deaths | Male | 30-34 years | 2803.82(-289.00，6004.22) | 0.92(-0.09，1.97) |
| Deaths | Female | 30-34 years | 1202.21(-100.96，2597.62) | 0.40(-0.03，0.87) |
| Deaths | Male | 35-39 years | 5264.88(-569.83，11204.57) | 1.86(-0.20，3.96) |
| Deaths | Female | 35-39 years | 2326.62(-194.76，4852.05) | 0.84(-0.07，1.75) |
| Deaths | Male | 40-44 years | 10961.36(-1261.97，23253.98) | 4.35(-0.50，9.22) |
| Deaths | Female | 40-44 years | 4633.93(-428.28，9932.69) | 1.87(-0.17，4.00) |
| Deaths | Male | 45-49 years | 19172.06(-2284.08，39892.16) | 8.06(-0.96，16.77) |
| Deaths | Female | 45-49 years | 8840.59(-860.69，19137.74) | 3.75(-0.37，8.12) |
| Deaths | Male | 50-54 years | 35503.07(-4335.61，73865.64) | 15.99(-1.95，33.28) |
| Deaths | Female | 50-54 years | 16048.09(-1647.38，33751.35) | 7.20(-0.74，15.14) |
| Deaths | Male | 55-59 years | 54862.43(-6854.37，113467.20) | 28.17(-3.52，58.27) |
| Deaths | Female | 55-59 years | 28469.74(-3103.86，59759.75) | 14.16(-1.54，29.73) |
| Deaths | Male | 60-64 years | 74358.13(-9663.32，153266.27) | 47.81(-6.21，98.54) |
| Deaths | Female | 60-64 years | 39750.23(-4578.13，84460.18) | 24.16(-2.78，51.34) |
| DALYs | Male | 95+ years | 110345.35(-12311.40，218553.86) | 7297.77(-814.22，14454.22) |
| DALYs | Female | 95+ years | 234932.16(-22975.64，486917.24) | 5965.37(-583.39，12363.74) |
| DALYs | Male | 25-29 years | 81480.01(-7138.38，168306.84) | 27.40(-2.40，56.60) |
| DALYs | Female | 25-29 years | 42255.34(-2995.18，90705.42) | 14.52(-1.03，31.17) |
| DALYs | Male | 30-34 years | 171162.22(-17575.75，363072.22) | 56.02(-5.75，118.83) |
| DALYs | Female | 30-34 years | 77377.62(-6650.28，165678.53) | 25.88(-2.22，55.42) |
| DALYs | Male | 35-39 years | 292411.46(-31806.49，614455.47) | 103.30(-11.24，217.07) |
| DALYs | Female | 35-39 years | 135165.22(-11848.80，283542.92) | 48.66(-4.27，102.07) |
| DALYs | Male | 40-44 years | 547119.93(-63712.89，1155573.06) | 216.97(-25.27，458.27) |
| DALYs | Female | 40-44 years | 239989.10(-22803.81，509008.10) | 96.73(-9.19，205.17) |
| DALYs | Male | 45-49 years | 862005.63(-104086.39，1789596.71) | 362.40(-43.76，752.37) |
| DALYs | Female | 45-49 years | 408753.87(-40528.76，867949.82) | 173.46(-17.20，368.33) |
| DALYs | Male | 50-54 years | 1417596.14(-175884.74，2937417.21) | 638.61(-79.23，1323.28) |
| DALYs | Female | 50-54 years | 657226.87(-68540.56，1364657.10) | 294.80(-30.74，612.11) |
| DALYs | Male | 55-59 years | 1923716.04(-244106.78，3952653.54) | 987.92(-125.36，2029.87) |
| DALYs | Female | 55-59 years | 1015110.16(-112056.98，2113650.59) | 505.02(-55.75，1051.55) |
| DALYs | Male | 60-64 years | 2246083.15(-295191.26，4610129.27) | 1444.08(-189.79，2964.01) |
| DALYs | Female | 60-64 years | 1218141.82(-141285.82，2589869.09) | 740.46(-85.88，1574.28) |
| DALYs | Male | 85-89 years | 1093370.53(-127207.19，2181387.78) | 6337.40(-737.32，12643.78) |
| DALYs | Female | 85-89 years | 993400.86(-107966.73，2112911.73) | 3489.40(-379.24，7421.76) |
| DALYs | Male | 90-94 years | 447024.53(-50473.62，877309.11) | 7669.63(-865.98，15052.06) |
| DALYs | Female | 90-94 years | 545964.92(-56859.18，1099664.59) | 4526.74(-471.43，9117.62) |

ASR: Age-standardized rate. DALYs：Disability-Adjusted Life Years.

Supplementary Table 2: Decomposition analysis results of the burden of cardiovascular disease attributable to lead exposure.

| **Metric** | **group** | **Sex** | **OD** | **Aging** | **Population** | **EC** | **AP** | **PP** | **ECP** |
| --- | --- | --- | --- | --- | --- | --- | --- | --- | --- |
| Deaths | Global | Both | 679938.36 | 320384.88 | 653188.52 | -293635.04 | 47.12 | 96.07 | -43.19 |
| Deaths | Low-middle SDI | Both | 205120.01 | 50666.55 | 201557.22 | -47103.77 | 24.7 | 98.26 | -22.96 |
| Deaths | High SDI | Both | 3936.42 | 49853.82 | 40459.66 | -86377.06 | 1266.47 | 1027.83 | -2194.3 |
| Deaths | High-middle SDI | Both | 126542.04 | 93257.58 | 101539.99 | -68255.52 | 73.7 | 80.24 | -53.94 |
| Deaths | Low SDI | Both | 63827.42 | -2761.94 | 91370.92 | -24781.56 | -4.33 | 143.15 | -38.83 |
| Deaths | Middle SDI | Both | 280126.63 | 173211.78 | 262283.49 | -155368.64 | 61.83 | 93.63 | -55.46 |
| Deaths | Global | Male | 396859.99 | 202395.61 | 369657.84 | -175193.45 | 51 | 93.15 | -44.14 |
| Deaths | Low-middle SDI | Male | 114929.58 | 21504.7 | 110596.52 | -17171.64 | 18.71 | 96.23 | -14.94 |
| Deaths | High SDI | Male | 1495 | 30563.71 | 23893.31 | -52962.02 | 2044.39 | 1598.21 | -3542.6 |
| Deaths | High-middle SDI | Male | 70232.29 | 57504.5 | 57776.48 | -45048.68 | 81.88 | 82.26 | -64.14 |
| Deaths | Low SDI | Male | 31635.81 | -3372.44 | 49721.44 | -14713.19 | -10.66 | 157.17 | -46.51 |
| Deaths | Middle SDI | Male | 178379.77 | 101119.26 | 149035.4 | -71774.9 | 56.69 | 83.55 | -40.24 |
| Deaths | Global | Female | 283078.37 | 131370.05 | 283143.13 | -131434.81 | 46.41 | 100.02 | -46.43 |
| Deaths | Low-middle SDI | Female | 90190.42 | 27656.24 | 89986.04 | -27451.86 | 30.66 | 99.77 | -30.44 |
| Deaths | High SDI | Female | 2441.42 | 23235.89 | 17172.54 | -37967.01 | 951.74 | 703.38 | -1555.12 |
| Deaths | High-middle SDI | Female | 56309.75 | 41357.3 | 44285.16 | -29332.71 | 73.45 | 78.65 | -52.09 |
| Deaths | Low SDI | Female | 32191.61 | 289.83 | 41439.37 | -9537.59 | 0.9 | 128.73 | -29.63 |
| Deaths | Middle SDI | Female | 101746.86 | 73261.37 | 111679.19 | -83193.7 | 72 | 109.76 | -81.77 |
| DALYs | Global | Both | 10986857.37 | 5207028.07 | 14441146.01 | -8661316.71 | 47.39 | 131.44 | -78.83 |
| DALYs | High-middle SDI | Both | 1444455.53 | 1389146.89 | 2028431.73 | -1973123.08 | 96.17 | 140.43 | -136.6 |
| DALYs | Low-middle SDI | Both | 4048339.58 | 876091.75 | 4903384.65 | -1731136.82 | 21.64 | 121.12 | -42.76 |
| DALYs | Middle SDI | Both | 4385539.4 | 2969395.58 | 5847564.89 | -4431421.08 | 67.71 | 133.34 | -101.05 |
| DALYs | High SDI | Both | -232864.42 | 654701.48 | 735808.43 | -1623374.33 | -281.15 | -315.98 | 697.13 |
| DALYs | Low SDI | Both | 1336378.62 | -115673.78 | 2277644.04 | -825591.64 | -8.66 | 170.43 | -61.78 |
| DALYs | Global | Male | 6769712.06 | 3421681.01 | 8626134.82 | -5278103.77 | 50.54 | 127.42 | -77.97 |
| DALYs | High-middle SDI | Male | 861534.05 | 930020.62 | 1252561.91 | -1321048.47 | 107.95 | 145.39 | -153.34 |
| DALYs | Low-middle SDI | Male | 2391465.73 | 383279.76 | 2792324.56 | -784138.58 | 16.03 | 116.76 | -32.79 |
| DALYs | Middle SDI | Male | 2969619.64 | 1800855.55 | 3462754.05 | -2293989.96 | 60.64 | 116.61 | -77.25 |
| DALYs | High SDI | Male | -153580.96 | 445759.18 | 485432.94 | -1084773.07 | -290.24 | -316.08 | 706.32 |
| DALYs | Low SDI | Male | 698124.07 | -104592.38 | 1281063.24 | -478346.8 | -14.98 | 183.5 | -68.52 |
| DALYs | Global | Female | 4217145.31 | 1981811.23 | 5794546.32 | -3559212.24 | 46.99 | 137.4 | -84.4 |
| DALYs | High-middle SDI | Female | 582921.48 | 548469.7 | 790048.24 | -755596.46 | 94.09 | 135.53 | -129.62 |
| DALYs | Low-middle SDI | Female | 1656873.85 | 462038.61 | 2078620.72 | -883785.48 | 27.89 | 125.45 | -53.34 |
| DALYs | Middle SDI | Female | 1415919.76 | 1185556.29 | 2337973.86 | -2107610.38 | 83.73 | 165.12 | -148.85 |
| DALYs | High SDI | Female | -79283.47 | 266173.62 | 267117.42 | -612574.5 | -335.72 | -336.91 | 772.64 |
| DALYs | Low SDI | Female | 638254.55 | -19084.24 | 989933.64 | -332594.85 | -2.99 | 155.1 | -52.11 |

**OD**: Overall Difference; **EC**: Epidemiological Change; **AP**: Aging Percentage; **PP**: Population Percentage;

**ECP**: Epidemiological Change Percentage. **DALYs**：Disability-Adjusted Life Years.

Supplementary Table 3: Results of the frontier analysis of the burden of cardiovascular disease attributable to

lead exposure in 204 countries in 2021.

|  |  | **Deaths** | | | **DALYs** | | |
| --- | --- | --- | --- | --- | --- | --- | --- |
| **Location** | **SDI** | **Value** | **Frontier** | **eff_diff** | **Value** | **Frontier** | **eff_diff** |
| China | 0.722 | 26.21 | 3.64 | 22.57 | 441.55 | 72.82 | 368.73 |
| Democratic People's Republic of Korea | 0.570 | 29.39 | 7.20 | 22.18 | 580.31 | 151.90 | 428.41 |
| Taiwan (Province of China) | 0.875 | 6.83 | 2.34 | 4.50 | 133.73 | 40.51 | 93.22 |
| Cambodia | 0.474 | 31.50 | 14.45 | 17.05 | 617.78 | 284.50 | 333.29 |
| Indonesia | 0.657 | 29.71 | 4.41 | 25.30 | 594.15 | 88.76 | 505.39 |
| Lao People's Democratic Republic | 0.489 | 38.93 | 14.36 | 24.57 | 786.38 | 282.57 | 503.80 |
| Malaysia | 0.743 | 12.86 | 3.53 | 9.34 | 270.41 | 69.15 | 201.26 |
| Maldives | 0.651 | 11.47 | 4.49 | 6.97 | 209.66 | 90.33 | 119.33 |
| Myanmar | 0.534 | 29.84 | 7.32 | 22.52 | 610.62 | 157.91 | 452.71 |
| Philippines | 0.651 | 17.69 | 4.49 | 13.20 | 383.53 | 90.31 | 293.23 |
| Sri Lanka | 0.702 | 8.53 | 3.78 | 4.75 | 160.76 | 76.62 | 84.13 |
| Thailand | 0.683 | 4.53 | 4.20 | 0.34 | 99.84 | 85.40 | 14.44 |
| Timor-Leste | 0.445 | 37.47 | 14.47 | 22.99 | 735.52 | 284.11 | 451.41 |
| Viet Nam | 0.628 | 18.79 | 4.78 | 14.01 | 366.76 | 97.30 | 269.46 |
| Fiji | 0.675 | 10.91 | 4.21 | 6.71 | 212.34 | 85.12 | 127.22 |
| Kiribati | 0.527 | 13.46 | 7.30 | 6.16 | 289.48 | 159.55 | 129.93 |
| Marshall Islands | 0.574 | 18.30 | 7.22 | 11.08 | 368.06 | 150.27 | 217.79 |
| Micronesia (Federated States of) | 0.588 | 16.23 | 6.05 | 10.18 | 345.86 | 125.74 | 220.12 |
| Papua New Guinea | 0.418 | 14.42 | 14.42 | 0.00 | 280.71 | 280.71 | 0.00 |
| Samoa | 0.593 | 11.42 | 5.82 | 5.60 | 222.04 | 115.69 | 106.35 |
| Solomon Islands | 0.429 | 32.68 | 14.48 | 18.21 | 645.51 | 283.61 | 361.90 |
| Tonga | 0.626 | 6.29 | 4.85 | 1.43 | 122.19 | 96.99 | 25.20 |
| Vanuatu | 0.473 | 24.76 | 14.48 | 10.28 | 520.48 | 284.02 | 236.46 |
| Armenia | 0.702 | 14.55 | 3.80 | 10.74 | 247.40 | 77.10 | 170.30 |
| Azerbaijan | 0.695 | 19.45 | 3.84 | 15.62 | 337.06 | 78.15 | 258.91 |
| Georgia | 0.732 | 24.62 | 3.58 | 21.04 | 469.76 | 70.46 | 399.30 |
| Kazakhstan | 0.725 | 12.85 | 3.66 | 9.19 | 218.91 | 71.01 | 147.90 |
| Kyrgyzstan | 0.604 | 20.91 | 5.59 | 15.32 | 376.69 | 108.35 | 268.34 |
| Mongolia | 0.618 | 22.05 | 4.99 | 17.06 | 397.45 | 99.26 | 298.20 |
| Tajikistan | 0.542 | 30.84 | 7.35 | 23.49 | 534.49 | 158.18 | 376.31 |
| Turkmenistan | 0.682 | 21.00 | 4.18 | 16.82 | 401.84 | 84.16 | 317.68 |
| Uzbekistan | 0.663 | 20.37 | 4.32 | 16.05 | 364.91 | 86.75 | 278.16 |
| Albania | 0.707 | 18.23 | 3.76 | 14.47 | 279.31 | 74.40 | 204.91 |
| Bosnia and Herzegovina | 0.723 | 17.92 | 3.62 | 14.30 | 308.16 | 72.05 | 236.11 |
| Bulgaria | 0.768 | 28.15 | 3.21 | 24.94 | 476.51 | 63.98 | 412.53 |
| Croatia | 0.798 | 9.79 | 2.80 | 6.99 | 153.10 | 50.86 | 102.24 |
| Czechia | 0.828 | 5.96 | 2.59 | 3.37 | 98.66 | 46.79 | 51.87 |
| Hungary | 0.791 | 10.61 | 2.94 | 7.67 | 194.28 | 54.20 | 140.08 |
| North Macedonia | 0.751 | 25.60 | 3.47 | 22.14 | 384.73 | 67.19 | 317.55 |
| Montenegro | 0.796 | 15.28 | 2.80 | 12.48 | 238.84 | 51.97 | 186.88 |
| Poland | 0.812 | 9.96 | 2.53 | 7.43 | 172.72 | 45.55 | 127.16 |
| Romania | 0.768 | 15.54 | 3.23 | 12.30 | 265.35 | 63.91 | 201.44 |
| Serbia | 0.792 | 15.69 | 2.88 | 12.81 | 253.76 | 54.11 | 199.65 |
| Slovakia | 0.811 | 9.31 | 2.56 | 6.75 | 161.64 | 46.49 | 115.14 |
| Slovenia | 0.842 | 4.03 | 2.51 | 1.52 | 62.52 | 46.47 | 16.05 |
| Belarus | 0.784 | 14.53 | 2.98 | 11.54 | 273.27 | 55.37 | 217.90 |
| Estonia | 0.845 | 9.50 | 2.54 | 6.96 | 150.17 | 46.34 | 103.83 |
| Latvia | 0.831 | 8.65 | 2.61 | 6.04 | 156.35 | 46.06 | 110.29 |
| Lithuania | 0.856 | 7.88 | 2.42 | 5.46 | 138.55 | 45.91 | 92.64 |
| Republic of Moldova | 0.732 | 14.66 | 3.54 | 11.12 | 281.63 | 70.08 | 211.55 |
| Russian Federation | 0.809 | 10.29 | 2.62 | 7.66 | 197.83 | 47.81 | 150.02 |
| Ukraine | 0.761 | 11.89 | 3.27 | 8.62 | 205.90 | 63.97 | 141.93 |
| Brunei Darussalam | 0.810 | 9.72 | 2.55 | 7.17 | 167.51 | 46.36 | 121.15 |
| Republic of Korea | 0.887 | 5.31 | 2.34 | 2.97 | 35.06 | 35.06 | 0.00 |
| Singapore | 0.856 | 5.59 | 2.44 | 3.15 | 83.96 | 42.36 | 41.60 |
| Australia | 0.844 | 4.59 | 2.59 | 2.00 | 107.68 | 43.12 | 64.56 |
| New Zealand | 0.849 | 5.32 | 2.51 | 2.81 | 75.53 | 45.88 | 29.65 |
| Andorra | 0.869 | 2.70 | 2.48 | 0.22 | 90.93 | 46.71 | 44.22 |
| Austria | 0.854 | 4.57 | 2.51 | 2.06 | 43.90 | 39.66 | 4.23 |
| Belgium | 0.854 | 5.04 | 2.43 | 2.61 | 70.15 | 44.77 | 25.38 |
| Cyprus | 0.836 | 11.07 | 2.57 | 8.50 | 87.61 | 46.19 | 41.42 |
| Denmark | 0.896 | 2.89 | 2.24 | 0.65 | 150.17 | 46.41 | 103.76 |
| Finland | 0.860 | 2.83 | 2.42 | 0.40 | 49.38 | 42.97 | 6.41 |
| France | 0.838 | 3.09 | 2.55 | 0.55 | 46.67 | 43.46 | 3.21 |
| Germany | 0.903 | 3.37 | 2.37 | 1.01 | 51.97 | 45.53 | 6.43 |
| Greece | 0.792 | 8.96 | 2.90 | 6.06 | 56.08 | 42.21 | 13.86 |
| Iceland | 0.876 | 3.50 | 2.43 | 1.07 | 148.82 | 52.45 | 96.36 |
| Ireland | 0.874 | 3.61 | 2.35 | 1.26 | 55.04 | 42.38 | 12.66 |
| Israel | 0.809 | 2.17 | 2.17 | 0.00 | 58.50 | 41.61 | 16.89 |
| Italy | 0.806 | 7.14 | 2.73 | 4.41 | 37.70 | 37.70 | 0.00 |
| Luxembourg | 0.884 | 3.24 | 2.32 | 0.92 | 98.36 | 50.76 | 47.60 |
| Malta | 0.802 | 10.11 | 2.72 | 7.39 | 50.43 | 39.98 | 10.44 |
| Netherlands | 0.888 | 2.97 | 2.41 | 0.57 | 171.60 | 50.64 | 120.96 |
| Norway | 0.916 | 2.59 | 2.31 | 0.29 | 46.85 | 41.44 | 5.41 |
| Portugal | 0.744 | 7.90 | 3.48 | 4.42 | 43.97 | 40.24 | 3.73 |
| Spain | 0.769 | 5.17 | 3.15 | 2.02 | 125.67 | 69.03 | 56.64 |
| Sweden | 0.887 | 2.46 | 2.27 | 0.20 | 84.68 | 62.91 | 21.77 |
| Switzerland | 0.933 | 4.24 | 2.28 | 1.96 | 40.01 | 39.36 | 0.65 |
| United Kingdom | 0.859 | 3.03 | 2.46 | 0.56 | 61.22 | 40.80 | 20.42 |
| Argentina | 0.723 | 6.41 | 3.65 | 2.76 | 52.52 | 45.79 | 6.73 |
| Chile | 0.772 | 2.68 | 2.68 | 0.00 | 117.56 | 71.50 | 46.06 |
| Uruguay | 0.719 | 8.77 | 3.67 | 5.11 | 51.51 | 51.51 | 0.00 |
| Canada | 0.873 | 2.44 | 2.22 | 0.23 | 164.65 | 72.17 | 92.47 |
| United States of America | 0.862 | 5.10 | 2.44 | 2.66 | 45.74 | 41.42 | 4.32 |
| Antigua and Barbuda | 0.750 | 17.25 | 3.45 | 13.80 | 96.67 | 43.16 | 53.51 |
| Bahamas | 0.805 | 13.34 | 2.73 | 10.61 | 290.62 | 68.29 | 222.33 |
| Barbados | 0.747 | 9.12 | 3.47 | 5.65 | 251.31 | 50.62 | 200.69 |
| Belize | 0.610 | 12.16 | 5.24 | 6.92 | 155.66 | 67.46 | 88.20 |
| Cuba | 0.669 | 18.94 | 4.27 | 14.67 | 225.90 | 106.27 | 119.63 |
| Dominica | 0.747 | 19.97 | 3.49 | 16.48 | 345.73 | 85.84 | 259.89 |
| Dominican Republic | 0.619 | 29.28 | 5.00 | 24.28 | 335.58 | 67.68 | 267.91 |
| Grenada | 0.669 | 23.40 | 4.26 | 19.14 | 575.74 | 98.91 | 476.83 |
| Guyana | 0.651 | 41.62 | 4.49 | 37.13 | 424.14 | 86.32 | 337.82 |
| Haiti | 0.448 | 65.29 | 14.48 | 50.81 | 815.33 | 90.27 | 725.06 |
| Jamaica | 0.683 | 19.98 | 4.10 | 15.88 | 1279.31 | 284.45 | 994.86 |
| Saint Lucia | 0.673 | 19.91 | 4.23 | 15.68 | 363.44 | 83.39 | 280.04 |
| Saint Vincent and the Grenadines | 0.637 | 37.50 | 4.68 | 32.82 | 330.15 | 86.33 | 243.81 |
| Suriname | 0.634 | 20.41 | 4.61 | 15.80 | 614.46 | 93.05 | 521.40 |
| Trinidad and Tobago | 0.769 | 8.33 | 3.19 | 5.14 | 402.18 | 93.54 | 308.64 |
| Bolivia (Plurinational State of) | 0.599 | 17.51 | 5.68 | 11.83 | 158.31 | 62.73 | 95.58 |
| Ecuador | 0.661 | 10.34 | 4.34 | 6.00 | 320.33 | 116.06 | 204.27 |
| Peru | 0.662 | 6.14 | 4.33 | 1.80 | 166.53 | 86.40 | 80.12 |
| Colombia | 0.655 | 9.24 | 4.40 | 4.84 | 124.94 | 86.10 | 38.83 |
| Costa Rica | 0.700 | 8.23 | 3.77 | 4.45 | 160.38 | 89.38 | 71.00 |
| El Salvador | 0.564 | 17.46 | 7.34 | 10.12 | 150.08 | 75.46 | 74.63 |
| Guatemala | 0.540 | 21.90 | 7.33 | 14.57 | 325.53 | 158.26 | 167.27 |
| Honduras | 0.513 | 50.05 | 7.69 | 42.36 | 366.92 | 158.41 | 208.51 |
| Mexico | 0.665 | 14.86 | 4.36 | 10.50 | 887.73 | 165.33 | 722.40 |
| Nicaragua | 0.524 | 15.41 | 7.37 | 8.05 | 253.35 | 87.08 | 166.27 |
| Panama | 0.709 | 9.41 | 3.70 | 5.71 | 255.93 | 161.16 | 94.77 |
| Venezuela (Bolivarian Republic of) | 0.597 | 21.82 | 5.64 | 16.18 | 170.64 | 73.82 | 96.82 |
| Brazil | 0.653 | 10.68 | 4.40 | 6.28 | 417.06 | 114.89 | 302.17 |
| Paraguay | 0.636 | 13.73 | 4.65 | 9.08 | 211.10 | 88.38 | 122.72 |
| Algeria | 0.660 | 39.73 | 4.42 | 35.31 | 253.10 | 94.55 | 158.55 |
| Bahrain | 0.753 | 18.50 | 3.41 | 15.08 | 596.18 | 88.33 | 507.85 |
| Egypt | 0.607 | 87.60 | 5.41 | 82.19 | 288.75 | 66.40 | 222.35 |
| Iran (Islamic Republic of) | 0.697 | 38.05 | 3.75 | 34.30 | 1559.71 | 105.14 | 1454.57 |
| Iraq | 0.663 | 39.87 | 4.35 | 35.52 | 652.02 | 76.07 | 575.95 |
| Jordan | 0.725 | 20.42 | 3.63 | 16.79 | 713.94 | 87.11 | 626.83 |
| Kuwait | 0.847 | 10.98 | 2.54 | 8.45 | 350.69 | 71.80 | 278.88 |
| Lebanon | 0.745 | 9.75 | 3.51 | 6.24 | 196.22 | 47.62 | 148.60 |
| Libya | 0.726 | 27.05 | 3.62 | 23.43 | 160.96 | 69.32 | 91.64 |
| Morocco | 0.563 | 36.53 | 7.35 | 29.18 | 488.77 | 71.55 | 417.22 |
| Palestine | 0.631 | 43.27 | 4.65 | 38.61 | 640.24 | 157.88 | 482.36 |
| Oman | 0.773 | 27.10 | 3.05 | 24.05 | 670.76 | 93.67 | 577.09 |
| Qatar | 0.847 | 9.75 | 2.51 | 7.23 | 456.32 | 59.35 | 396.97 |
| Saudi Arabia | 0.815 | 34.30 | 2.60 | 31.70 | 158.44 | 45.98 | 112.45 |
| Syrian Arab Republic | 0.623 | 55.50 | 4.95 | 50.55 | 646.94 | 46.61 | 600.34 |
| Tunisia | 0.682 | 31.16 | 4.14 | 27.03 | 930.84 | 98.28 | 832.56 |
| Turkey | 0.713 | 16.34 | 3.76 | 12.58 | 510.28 | 83.77 | 426.50 |
| United Arab Emirates | 0.849 | 17.77 | 2.50 | 15.27 | 256.58 | 74.05 | 182.53 |
| Yemen | 0.450 | 87.19 | 14.48 | 72.71 | 286.36 | 46.19 | 240.17 |
| Afghanistan | 0.337 | 98.11 | 15.36 | 82.75 | 1628.04 | 284.49 | 1343.56 |
| Bangladesh | 0.492 | 37.18 | 11.16 | 26.02 | 1948.18 | 326.69 | 1621.50 |
| Bhutan | 0.473 | 30.54 | 14.46 | 16.08 | 682.60 | 242.28 | 440.31 |
| India | 0.575 | 27.27 | 7.17 | 20.10 | 560.09 | 285.06 | 275.03 |
| Nepal | 0.433 | 41.49 | 14.49 | 27.00 | 558.12 | 147.59 | 410.53 |
| Pakistan | 0.504 | 37.25 | 8.63 | 28.62 | 771.75 | 284.56 | 487.19 |
| Angola | 0.454 | 30.77 | 14.47 | 16.29 | 735.16 | 187.03 | 548.14 |
| Central African Republic | 0.309 | 48.66 | 15.61 | 33.05 | 585.13 | 283.53 | 301.60 |
| Congo | 0.583 | 25.46 | 7.01 | 18.45 | 994.13 | 347.75 | 646.38 |
| Democratic Republic of the Congo | 0.383 | 33.04 | 15.36 | 17.68 | 482.08 | 142.88 | 339.20 |
| Equatorial Guinea | 0.658 | 22.36 | 4.42 | 17.94 | 630.28 | 321.02 | 309.26 |
| Gabon | 0.635 | 21.08 | 4.63 | 16.45 | 411.60 | 88.68 | 322.93 |
| Burundi | 0.289 | 30.34 | 16.99 | 13.35 | 384.47 | 93.30 | 291.17 |
| Comoros | 0.476 | 23.08 | 14.45 | 8.63 | 588.24 | 351.21 | 237.03 |
| Djibouti | 0.488 | 26.60 | 14.37 | 12.23 | 418.44 | 283.76 | 134.68 |
| Eritrea | 0.404 | 35.18 | 15.19 | 19.99 | 497.28 | 282.40 | 214.89 |
| Ethiopia | 0.359 | 27.79 | 15.36 | 12.44 | 649.23 | 299.34 | 349.89 |
| Kenya | 0.524 | 18.37 | 7.40 | 10.96 | 511.97 | 326.23 | 185.74 |
| Madagascar | 0.400 | 39.07 | 15.28 | 23.79 | 326.28 | 160.31 | 165.97 |
| Malawi | 0.385 | 29.79 | 15.36 | 14.43 | 755.80 | 305.28 | 450.51 |
| Mauritius | 0.718 | 9.50 | 3.64 | 5.85 | 578.27 | 320.43 | 257.84 |
| Mozambique | 0.326 | 48.92 | 15.36 | 33.56 | 193.71 | 73.88 | 119.82 |
| Rwanda | 0.436 | 25.98 | 14.47 | 11.51 | 960.27 | 327.07 | 633.19 |
| Seychelles | 0.730 | 10.57 | 3.58 | 6.99 | 463.60 | 283.96 | 179.64 |
| Somalia | 0.078 | 51.92 | 51.92 | 0.00 | 198.93 | 70.14 | 128.80 |
| United Republic of Tanzania | 0.447 | 19.36 | 14.49 | 4.87 | 1066.65 | 1066.65 | 0.00 |
| Uganda | 0.423 | 21.77 | 14.48 | 7.29 | 349.99 | 283.98 | 66.01 |
| Zambia | 0.506 | 35.99 | 8.86 | 27.13 | 422.65 | 284.38 | 138.27 |
| Botswana | 0.643 | 24.05 | 4.55 | 19.50 | 634.84 | 185.34 | 449.50 |
| Lesotho | 0.510 | 46.83 | 8.09 | 38.75 | 455.89 | 91.91 | 363.99 |
| Namibia | 0.618 | 27.36 | 4.94 | 22.41 | 974.29 | 169.59 | 804.70 |
| South Africa | 0.680 | 15.69 | 4.16 | 11.53 | 520.47 | 98.18 | 422.28 |
| Eswatini | 0.585 | 32.88 | 7.00 | 25.87 | 301.36 | 84.45 | 216.91 |
| Zimbabwe | 0.474 | 30.79 | 14.46 | 16.33 | 682.51 | 141.95 | 540.56 |
| Benin | 0.373 | 22.12 | 15.35 | 6.77 | 643.03 | 284.83 | 358.19 |
| Burkina Faso | 0.285 | 37.99 | 16.78 | 21.21 | 432.41 | 326.19 | 106.22 |
| Cameroon | 0.480 | 29.29 | 14.46 | 14.84 | 757.08 | 356.25 | 400.83 |
| Cabo Verde | 0.534 | 18.42 | 7.33 | 11.08 | 598.91 | 283.89 | 315.02 |
| Chad | 0.240 | 44.94 | 24.15 | 20.79 | 342.06 | 158.10 | 183.96 |
| Côte d'Ivoire | 0.426 | 28.13 | 14.47 | 13.66 | 929.62 | 516.06 | 413.56 |
| Gambia | 0.410 | 36.98 | 14.90 | 22.09 | 565.82 | 284.32 | 281.51 |
| Ghana | 0.565 | 20.59 | 7.28 | 13.30 | 740.54 | 292.64 | 447.91 |
| Guinea | 0.336 | 37.55 | 15.35 | 22.20 | 416.88 | 157.47 | 259.41 |
| Guinea-Bissau | 0.353 | 46.48 | 15.37 | 31.12 | 757.92 | 326.19 | 431.73 |
| Liberia | 0.352 | 33.23 | 15.34 | 17.89 | 976.19 | 326.28 | 649.91 |
| Mali | 0.269 | 30.14 | 24.14 | 6.01 | 670.71 | 326.21 | 344.49 |
| Mauritania | 0.499 | 20.69 | 10.39 | 10.30 | 631.71 | 500.62 | 131.08 |
| Niger | 0.168 | 36.43 | 30.93 | 5.50 | 397.30 | 194.41 | 202.89 |
| Nigeria | 0.503 | 16.46 | 9.85 | 6.61 | 736.68 | 687.66 | 49.02 |
| Sao Tome and Principe | 0.505 | 20.20 | 8.72 | 11.48 | 315.56 | 206.54 | 109.01 |
| Senegal | 0.408 | 24.80 | 15.06 | 9.73 | 402.18 | 183.94 | 218.25 |
| Sierra Leone | 0.359 | 30.16 | 15.34 | 14.82 | 477.11 | 295.91 | 181.20 |
| Togo | 0.409 | 28.14 | 15.04 | 13.09 | 624.44 | 326.60 | 297.84 |
| American Samoa | 0.724 | 5.27 | 3.63 | 1.65 | 578.90 | 295.97 | 282.92 |
| Bermuda | 0.821 | 4.44 | 2.57 | 1.87 | 105.69 | 71.94 | 33.75 |
| Cook Islands | 0.779 | 6.88 | 3.08 | 3.80 | 74.80 | 49.02 | 25.79 |
| Greenland | 0.826 | 4.78 | 2.60 | 2.17 | 127.36 | 57.38 | 69.98 |
| Guam | 0.804 | 2.80 | 2.54 | 0.26 | 92.32 | 47.19 | 45.14 |
| Monaco | 0.908 | 3.76 | 2.35 | 1.41 | 69.76 | 45.97 | 23.79 |
| Nauru | 0.625 | 14.58 | 4.81 | 9.77 | 61.42 | 40.29 | 21.13 |
| Niue | 0.726 | 7.54 | 3.62 | 3.92 | 315.06 | 96.81 | 218.25 |
| Northern Mariana Islands | 0.772 | 3.74 | 2.95 | 0.79 | 147.36 | 71.75 | 75.61 |
| Palau | 0.754 | 5.73 | 3.37 | 2.36 | 73.61 | 61.85 | 11.76 |
| Puerto Rico | 0.826 | 4.27 | 2.59 | 1.67 | 114.34 | 66.37 | 47.97 |
| Saint Kitts and Nevis | 0.755 | 18.20 | 3.35 | 14.85 | 81.76 | 48.79 | 32.97 |
| San Marino | 0.888 | 1.88 | 1.88 | 0.00 | 308.13 | 65.83 | 242.30 |
| Tokelau | 0.686 | 8.56 | 4.06 | 4.50 | 162.78 | 81.07 | 81.71 |
| Tuvalu | 0.577 | 17.08 | 7.18 | 9.90 | 339.60 | 147.54 | 192.06 |
| United States Virgin Islands | 0.822 | 7.21 | 2.57 | 4.64 | 121.48 | 48.77 | 72.71 |
| South Sudan | 0.278 | 29.04 | 19.89 | 9.15 | 566.54 | 430.10 | 136.44 |
| Sudan | 0.542 | 57.62 | 7.34 | 50.28 | 1100.86 | 157.84 | 943.02 |

**eff_diff : Effective Difference.**

Supplementary Table 4: The top 5 Increase and bottom 20 Decrease countries in the frontier analysis of the burden of cardiovascular disease attributable to lead exposure.

| **Metric** | **location** | **Group** | **Value** | **SDI** | **Frontier** | **eff_diff** | **Trend** |
| --- | --- | --- | --- | --- | --- | --- | --- |
| Deaths | Afghanistan | Black | 98.112 | 0.337 | 15.362 | 82.750 | Decrease |
| Deaths | Egypt | Black | 87.602 | 0.607 | 5.410 | 82.192 | Decrease |
| Deaths | Yemen | Black | 87.188 | 0.450 | 14.479 | 72.710 | Decrease |
| Deaths | Haiti | Black | 65.291 | 0.448 | 14.478 | 50.813 | Decrease |
| Deaths | Syrian Arab Republic | Black | 55.496 | 0.623 | 4.948 | 50.548 | Decrease |
| Deaths | Sudan | Black | 57.618 | 0.542 | 7.341 | 50.277 | Decrease |
| Deaths | Honduras | Black | 50.054 | 0.513 | 7.691 | 42.363 | Increase |
| Deaths | Lesotho | Black | 46.833 | 0.510 | 8.086 | 38.747 | Increase |
| Deaths | Palestine | Black | 43.269 | 0.631 | 4.655 | 38.614 | Decrease |
| Deaths | Guyana | Black | 41.618 | 0.651 | 4.491 | 37.126 | Decrease |
| Deaths | Iraq | Black | 39.870 | 0.663 | 4.354 | 35.517 | Decrease |
| Deaths | Algeria | Black | 39.732 | 0.660 | 4.422 | 35.310 | Decrease |
| Deaths | Iran (Islamic Republic of) | Black | 38.055 | 0.697 | 3.755 | 34.300 | Decrease |
| Deaths | Mozambique | Black | 48.922 | 0.326 | 15.364 | 33.558 | Increase |
| Deaths | Central African Republic | Black | 48.663 | 0.309 | 15.609 | 33.054 | Increase |
| Deaths | Papua New Guinea | Blue | 14.419 | 0.418 | 14.419 | 0.000 | Decrease |
| Deaths | Somalia | Blue | 51.916 | 0.078 | 51.916 | 0.000 | Decrease |
| Deaths | United Republic of Tanzania | Blue | 19.361 | 0.447 | 14.493 | 4.868 | Decrease |
| Deaths | Niger | Blue | 36.430 | 0.168 | 30.929 | 5.500 | Increase |
| Deaths | Mali | Blue | 30.144 | 0.269 | 24.138 | 6.006 | Decrease |
| Deaths | Lithuania | Red | 7.881 | 0.856 | 2.424 | 5.457 | Decrease |
| Deaths | Taiwan (Province of China) | Red | 6.834 | 0.875 | 2.336 | 4.499 | Decrease |
| Deaths | Singapore | Red | 5.588 | 0.856 | 2.441 | 3.147 | Decrease |
| Deaths | Republic of Korea | Red | 5.311 | 0.887 | 2.345 | 2.966 | Decrease |
| Deaths | United States of America | Red | 5.097 | 0.862 | 2.441 | 2.657 | Decrease |
| DALYs | Afghanistan | Black | 1948.185 | 0.337 | 326.688 | 1621.496 | Decrease |
| DALYs | Egypt | Black | 1559.712 | 0.607 | 105.143 | 1454.569 | Decrease |
| DALYs | Yemen | Black | 1628.042 | 0.450 | 284.485 | 1343.557 | Decrease |
| DALYs | Haiti | Black | 1279.313 | 0.448 | 284.451 | 994.862 | Decrease |
| DALYs | Sudan | Black | 1100.859 | 0.542 | 157.842 | 943.017 | Decrease |
| DALYs | Syrian Arab Republic | Black | 930.841 | 0.623 | 98.284 | 832.557 | Decrease |
| DALYs | Lesotho | Black | 974.285 | 0.510 | 169.587 | 804.698 | Increase |
| DALYs | Guyana | Black | 815.333 | 0.651 | 90.269 | 725.064 | Decrease |
| DALYs | Honduras | Black | 887.732 | 0.513 | 165.332 | 722.399 | Increase |
| DALYs | Guinea-Bissau | Black | 976.194 | 0.353 | 326.283 | 649.911 | Decrease |
| DALYs | Central African Republic | Black | 994.128 | 0.309 | 347.746 | 646.382 | Decrease |
| DALYs | Mozambique | Black | 960.267 | 0.326 | 327.073 | 633.194 | Decrease |
| DALYs | Iraq | Black | 713.944 | 0.663 | 87.109 | 626.835 | Decrease |
| DALYs | Saudi Arabia | Black | 646.944 | 0.815 | 46.607 | 600.337 | Decrease |
| DALYs | Palestine | Black | 670.764 | 0.631 | 93.671 | 577.094 | Decrease |
| DALYs | Papua New Guinea | Blue | 280.715 | 0.418 | 280.715 | 0.000 | Decrease |
| DALYs | Somalia | Blue | 1066.648 | 0.078 | 1066.648 | 0.000 | Decrease |
| DALYs | Niger | Blue | 736.683 | 0.168 | 687.659 | 49.024 | Decrease |
| DALYs | United Republic of Tanzania | Blue | 349.993 | 0.447 | 283.984 | 66.009 | Decrease |
| DALYs | Benin | Blue | 432.411 | 0.373 | 326.191 | 106.220 | Decrease |
| DALYs | Taiwan (Province of China) | Red | 133.729 | 0.875 | 40.507 | 93.222 | Decrease |
| DALYs | Lithuania | Red | 138.547 | 0.856 | 45.906 | 92.641 | Decrease |
| DALYs | Singapore | Red | 107.678 | 0.856 | 43.120 | 64.558 | Decrease |
| DALYs | United States of America | Red | 96.674 | 0.862 | 43.160 | 53.514 | Decrease |
| DALYs | Republic of Korea | Red | 83.960 | 0.887 | 42.363 | 41.597 | Decrease |

SDI: Socio-demographic Index; DALYs: Disability-Adjusted Life Years.

Supplementary Table 5: Lead exposure-attributable burden of cardiovascular disease subtypes, 1990-2021

| Subtypes | Measure | Number 1990 | ASR 1990 | Number 2021 | ASR 2021 | EAPC(95% CI) |
| --- | --- | --- | --- | --- | --- | --- |
| Stroke | Deaths | 341294.06 (-45846.85, 759949.12) | 9.33 (-1.26, 20.82) | 556595.88 (-73988.03, 1225742.18) | 6.65 (-0.89, 14.63) | -1.23 (-1.40, -1.06) |
| Lower extremity peripheral arterial disease | Deaths | 142.81 (-20.53, 369.36) | 0.00 (0.00, 0.01) | 344.54 (-50.79, 900.30) | 0.00 (0.00, 0.01) | -0.28 (-0.47, -0.09) |
| Aortic aneurysm | Deaths | 487.04 (-63.82, 1304.50) | 0.01 (0.00, 0.04) | 1014.76 (-132.77, 2696.21) | 0.01 (0.00, 0.03) | -0.48 (-0.64, -0.32) |
| Hypertensive heart disease | Deaths | 174213.17 (6.92, 319338.91) | 5.03 (0.00, 9.21) | 318859.89 (5.13, 578584.98) | 3.92 (0.00, 7.11) | -0.72 (-0.82, -0.62) |
| Ischemic heart disease | Deaths | 277681.94 (-40555.95, 595671.21) | 7.91 (-1.15, 16.94) | 590370.03 (-83778.27, 1233628.18) | 7.11 (-1.01, 14.88) | -0.30 (-0.40, -0.20) |
| Atrial fibrillation and flutter | Deaths | 2480.89 (-357.17, 6298.29) | 0.09 (-0.01, 0.22) | 9053.18 (-1392.65, 22236.49) | 0.12 (-0.02, 0.29) | 0.90 (0.78, 1.01) |
| Stroke | DALYs | 8518064.73 (-1142372.12, 18888591.37) | 213.99 (-28.76, 475.41) | 12023066.18 (-1593054.62, 26565153.27) | 139.82 (-18.54, 308.86) | -1.51 (-1.67, -1.34) |
| Lower extremity peripheral arterial disease | DALYs | 4230.92 (-598.24, 11395.50) | 0.12 (-0.02, 0.31) | 8963.56 (-1277.69, 24169.43) | 0.11 (-0.02, 0.29) | -0.41 (-0.57, -0.26) |
| Aortic aneurysm | DALYs | 11146.84 (-1441.26, 29884.35) | 0.28 (-0.04, 0.76) | 20362.45 (-2634.57, 54019.71) | 0.24 (-0.03, 0.63) | -0.74 (-0.89, -0.59) |
| Hypertensive heart disease | DALYs | 3932980.41 (259.42, 7135509.25) | 102.12 (0.01, 185.41) | 5887523.49 (187.31, 10748875.34) | 69.93 (0.00, 127.40) | -1.20 (-1.29, -1.11) |
| Ischemic heart disease | DALYs | 6484403.92 (-947861.76, 14078198.14) | 166.11 (-24.29, 359.91) | 11854611.43 (-1668553.12, 24791275.33) | 138.57 (-19.52, 289.73) | -0.58 (-0.68, -0.47) |
| Atrial fibrillation and flutter | DALYs | 79858.19 (-10305.86, 205458.67) | 2.32 (-0.30, 5.95) | 223015.26 (-29502.69, 563551.70) | 2.70 (-0.36, 6.83) | 0.50 (0.39, 0.61) |

DALYs: Disability-Adjusted Life Years. ASR: Age-standardized rate.

Supplementary Table 6: ARIMA model prediction results of the burden of cardiovascular disease attributable to lead exposure (2021-2040, stratified by gender).

| Year | Both | | | Male | | | Female | | |
| --- | --- | --- | --- | --- | --- | --- | --- | --- | --- |
| 2021 | 17.816 | 17.816 | 17.816 | 23.490 | 23.490 | 23.490 | 13.417 | 13.417 | 13.417 |
| 2022 | 17.538 | 17.260 | 17.815 | 23.111 | 22.670 | 23.552 | 13.232 | 13.037 | 13.426 |
| 2023 | 17.283 | 16.749 | 17.818 | 22.775 | 21.949 | 23.600 | 13.047 | 12.666 | 13.429 |
| 2024 | 17.029 | 16.276 | 17.783 | 22.438 | 21.285 | 23.592 | 12.863 | 12.328 | 13.398 |
| 2025 | 16.775 | 15.809 | 17.742 | 22.102 | 20.630 | 23.574 | 12.679 | 11.998 | 13.360 |
| 2026 | 16.521 | 15.339 | 17.703 | 21.766 | 19.974 | 23.557 | 12.495 | 11.669 | 13.321 |
| 2027 | 16.267 | 14.864 | 17.669 | 21.429 | 19.311 | 23.547 | 12.311 | 11.338 | 13.284 |
| 2028 | 16.013 | 14.383 | 17.642 | 21.093 | 18.640 | 23.546 | 12.126 | 11.003 | 13.250 |
| 2029 | 15.759 | 13.895 | 17.622 | 20.756 | 17.958 | 23.555 | 11.942 | 10.665 | 13.219 |
| 2030 | 15.504 | 13.400 | 17.609 | 20.420 | 17.267 | 23.573 | 11.758 | 10.323 | 13.193 |
| 2031 | 15.250 | 12.897 | 17.604 | 20.084 | 16.565 | 23.603 | 11.574 | 9.977 | 13.170 |
| 2032 | 14.996 | 12.386 | 17.606 | 19.747 | 15.852 | 23.642 | 11.390 | 9.627 | 13.152 |
| 2033 | 14.742 | 11.869 | 17.615 | 19.411 | 15.129 | 23.692 | 11.205 | 9.272 | 13.139 |
| 2034 | 14.488 | 11.344 | 17.632 | 19.075 | 14.396 | 23.753 | 11.021 | 8.913 | 13.129 |
| 2035 | 14.234 | 10.811 | 17.656 | 18.738 | 13.653 | 23.824 | 10.837 | 8.550 | 13.124 |
| 2036 | 13.980 | 10.272 | 17.687 | 18.402 | 12.899 | 23.904 | 10.653 | 8.183 | 13.123 |
| 2037 | 13.725 | 9.726 | 17.725 | 18.065 | 12.136 | 23.995 | 10.468 | 7.811 | 13.126 |
| 2038 | 13.471 | 9.173 | 17.770 | 17.729 | 11.362 | 24.096 | 10.284 | 7.435 | 13.133 |
| 2039 | 13.217 | 8.613 | 17.822 | 17.393 | 10.579 | 24.206 | 10.100 | 7.056 | 13.144 |
| 2040 | 12.963 | 8.046 | 17.880 | 17.056 | 9.787 | 24.325 | 9.916 | 6.672 | 13.160 |

Supplementary Table 7: Results of Gender-stratified ARIMA Analysis and Stationarity Test on the Burden of cardiovascular disease attributable to lead exposure.

|  | **Metric** | **sex** | **p** | **d** | **q** | **KPSS_stat** | **KPSS_pval** | **LB_stat** | **LB_pval** |
| --- | --- | --- | --- | --- | --- | --- | --- | --- | --- |
| KPSS Level...1 | Death | Male | 0 | 2 | 2 | 0.829 | 0.01 | 9.992 | 0.266 |
| KPSS Level...2 | Death | Female | 0 | 2 | 2 | 0.880 | 0.01 | 7.021 | 0.534 |
| KPSS Level...3 | Death | Both | 0 | 2 | 2 | 0.856 | 0.01 | 10.046 | 0.262 |
| KPSS Level...1 | DALY | Male | 0 | 2 | 0 | 0.865 | 0.01 | 13.262 | 0.209 |
| KPSS Level...2 | DALY | Female | 0 | 2 | 0 | 0.890 | 0.01 | 6.636 | 0.759 |
| KPSS Level...3 | DALY | Both | 0 | 2 | 0 | 0.878 | 0.01 | 12.311 | 0.265 |

KPSS：Kwiatkowski-Phillips-Schmidt-Shin Test；KPSS_stat：KPSS Test Statistic；LB_stat：Ljung-Box Test Statistic； LB_pval：Ljung-Box Test p-value.
